# Field implementation of the sterile insect technique against *Aedes aegypti* in Recife, Brazil: operational challenges and impact of release frequency on vector dynamics

**DOI:** 10.1186/s40249-025-01393-7

**Published:** 2026-01-29

**Authors:** Aline Taiane Macedo, Danilo O. Carvalho, Maylen Gomez, Bianca Pires, Mirian Brito, Nilton Sousa, Aricia R. P. da Cruz, Helen Jamil Khoury, Jair F. Virginio

**Affiliations:** 1 Biofacility Moscamed Brasil, Sterile Aedes Production, Block D-13, Plot 15, São Francisco Industrial District, Juazeiro, Brazil; 2 Joint Food and Agriculture Organization & International Atomic Energy Agency Program of Nuclear Techniques in Food and Agriculture, Vienna, Austria; 3 Nuclear Energy Department, Federal University of Pernambuco, Recife, Brazil

**Keywords:** *Aedes aegypti*, Sterile insect technique, Mosquito suppression, Vector control, Population dynamics, Irradiation, Field trial

## Abstract

**Background:**

The sterile insect technique (SIT) is an environmentally friendly tool for suppressing *Aedes aegypti* populations. While promising in controlled settings, its application in large urban environments presents logistical and biological challenges. This trial focused on releasing sterile males, sent from a long-distance production facility to suppress the local mosquito population.

**Methods:**

Sterile males of *Ae. aegypti* were mass-reared, irradiated, and transported 712.2 km from a central facility to Recife, Brazil. Releases were performed once (SIT 1 ×) or twice per week (SIT 2 ×). Entomological indices—including eggs/trap per day (ETD), hatch rate, induced sterility, and adult female abundance—were monitored through ovitraps and BG-Sentinel traps. Data were analyzed using generalized linear mixed models (GLMMs) and Bayesian time-series modeling (CausalImpact).

**Results:**

Dose–response experiments established that pupae required 35 Gy and adults 65 Gy to achieve > 99% sterility, with no difference between gamma and X-ray sources. Adult sterilization was effective across 24–96 h post-emergence, facilitating operational flexibility. Handling and transport reduced flight ability by up to 35 percentage points, highlighting cumulative stress effects. In field trials, SIT 1 × yielded limited suppression, with ETD values remaining similar to or higher than those of the control. In contrast, SIT 2 × produced consistent suppression, reducing ETD by 39%, hatch rate by 33%, and female abundance by 51%.

**Conclusions:**

In this study, increasing the release frequency was essential to achieve significant model outcomes, representing varying degrees of mild suppression of *Ae. aegypti* in a complex urban setting. In Addition, male handling, chilling, and transport emphasize the need to reduce the exposure to these parameters by improving the protocols. These results highlight key areas for scaling SIT within integrated vector management strategies in tropical urban settings.

**Graphical Abstract:**

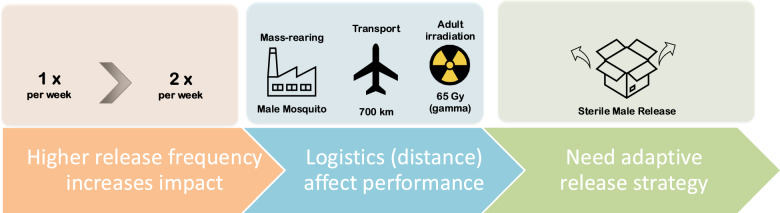

**Supplementary Information:**

The online version contains supplementary material available at 10.1186/s40249-025-01393-7.

## Background

Vector-borne diseases are a primary global health concern, especially in Brazil, where the *Aedes aegypti* mosquito transmits viruses causing dengue, Zika, and chikungunya [1, 2]. These illnesses impact public health and socioeconomic development [3]. In 2024, over 12.9 million dengue cases and 8135 deaths were reported in the Americas, with Brazil accounting for nearly half of the cases and over 73% of deaths [4]. In early 2025, Brazil reported 807,778 cases and 451 deaths. During the 2015/2016 Zika outbreak, Recife was heavily affected [5]. Limited access to vaccines and treatments, coupled with healthcare system challenges post-COVID-19, complicates disease management [6].

To prevent the spread of these arboviruses, new techniques are being developed to control *Ae. aegypti* mosquito populations. These should be integrated with traditional methods in vector management (IVM), as the conventional approaches have limited effectiveness due to issues like their application methods, resilience, development of resistant populations, and limited reach [7, 8]. The sterile insect technique (SIT) is an efficient method for controlling insect pests worldwide, including fruit flies, tsetse flies, screwworms, and moths, as well as vectors of diseases such as mosquitoes and tsetse flies [9–11]. SIT is an environmentally friendly way of managing insect populations that uses the principles of reproductive biology to suppress the population of a specific insect species [12]. The technique involves mass-rearing, sterilization by ionizing radiation, and field release of sterile males that mate with wild females, resulting in non-viable offspring and a decline in the target insect population over time. Although initially developed for agricultural pest control, SIT has recently shown promising results in managing vector populations [9, 11] in various settings and in reducing disease transmission in animals [10].

The SIT application requires releasing a vast number of sterile male insects into the target areas, with the number exceeding the wild male population. Therefore, mass-rearing of the insect species is essential for the technique to be effective. Knowledge of the target insect's biology, behavior, and nutrition is necessary to replicate the insect's life cycle under artificial conditions without affecting biological and behavioral parameters, such as fecundity, fertility, mating, longevity, sexual performance, and oviposition patterns, among others [12].

The quality of sterile males can be affected by biotic and abiotic factors throughout the workflow process, from production to the release of sterile males. Insects mass-reared for several generations may not have the same abilities as wild ones, and modifications in behavior may occur [13]. Even if “good quality” insects are produced, their behavior and sexual performance in the field can be affected by rearing, packaging, transporting, irradiation, and releasing procedures, compromising the SIT’s effectiveness. Therefore, it is necessary to establish physio-morphological and behavioral standards to monitor the quality of sterile males produced and release them with outstanding quality [14].

SIT continuously seeks to optimize methodologies for rearing, irradiating, handling, shipping, and releasing sterile males to promote operational cost reduction and ensure the quality of sterile males' adaptation to local mosquito strains in artificial rearing conditions [14]. At the same time, protocols should be developed to evaluate their survival, dispersal, and ability to reduce the wild population, allowing for their potential use in SIT. These steps are essential before the technology can be implemented in national and regional programs for integrated vector control [15, 16].

Ensuring proper sterility conditions demands access to an irradiation capacity and establishing a dose–response curve. This step is crucial for determining the minimum dose required to achieve the lowest residual fertility for a specific mosquito population, as unnecessary, more prolonged exposure (higher dose application) leads to a significant decrease in competitiveness, mating performance, or premature death, thereby precluding the use of SIT [17, 18].

The Brazilian authorities have initiated the process of evaluating SIT as a sustainable and cost-effective approach for controlling *Ae. aegypti*. Adopting SIT represents a promising shift in managing *Ae. aegypti* populations and curbing disease transmission dynamics. This technology will offer a viable alternative to conventional control methods and potentially reduce the burden of mosquito-borne diseases in the country [19].

This study reports on the feasibility and efficacy of employing radiation-induced SIT for *Ae. aegypti* control in a specific area of Recife, Brazil. The study investigates the scientific principles that underpin SIT, assesses its applicability within the urban context of Recife, and evaluates the potential benefits, challenges, and ethical considerations associated with its implementation.

## Methods

### Study site

**Fig. 1:**
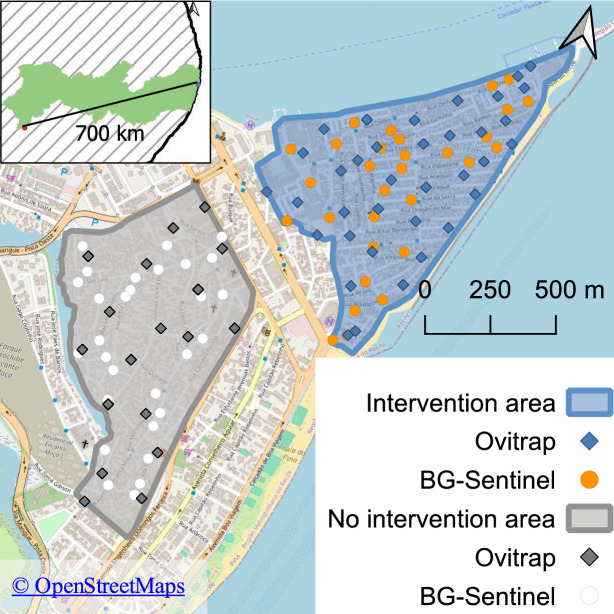
Intervention area in Brasilia Teimosa, with 60 ha (blue map), and no-intervention area in Pina, with 58 ha (grey map), with the positioning of the trapping network using ovitraps (blue and grey lozenges) and BG-Sentinels (orange and white dots). *BG* Biogents, Germany

The SIT pilot project had two study areas in Recife (Fig. 1): Brasilia Teimosa (BRT) was the intervention area, with 60 hectares (8°5′28.8" S, 34°53′14.5" W), and Pina (PIN) was the non-intervention area, with 58 hectares (8°5′4.7" S, 34°52′49.8" W). Both neighborhoods are coastal communities with similar population densities, ranging from approximately 18,000 to 29,000 residents each, and share comparable social determinants, as well as environmental and climatic conditions. These include limited access to basic sanitation infrastructure, mixed-income housing conditions typical of urban coastal areas in Northeast Brazil, and proximity to both informal settlements and more established residential areas.

### Baseline survey

Before the first release, the study area underwent an initial intervention phase aimed at eliminating the mosquito population. This phase involved applying pyriproxfen (PPF) from September 2, 2019, to March 3, 2020, and *Bacillus thuringiensis israelensis* (Bti) from March 25 to September 9, 2020, followed by another PPF application from September 9 to October 23, 2020. For Bti, the task was carried out not only in ovitraps but also at any potential and identified breeding sites, before the release of sterile males. And PPF were distributed at dissemination stations. During the sterile male release period, five insecticide applications were made between the intervention and non-intervention areas, always conducted at both sites.

#### Egg monitoring with ovitraps

Before the release of sterile males, the mosquito eggs baseline data were collected during 2 periods: the first one established with the deployment of 34 ovitraps in the intervention area and 18 in the non-intervention area, serviced twice a month (every 2 weeks) by the Health Secretary’s team, between 2017 and 2019. During this period, each ovitrap was treated with Bti (larvicide 0.21 g of the product per 0.5 L) to prevent it from becoming a breeding site between sampling intervals. After 2019, these ovitraps became part of the monitoring network for the SIT evaluation project. As a result, the servicing and inspection processes were transferred to Biofábrica Moscamed Brasil's responsibility. The traps were georeferenced, and the collection schedule was changed to a weekly collection, making the use of larvicide unnecessary and allowing egg hatching. After Biofábrica Moscamed Brasil took over the monitoring network of ovitraps in 2019, the paddles were analyzed to obtain the number of eggs/trap per day (ETD) and to estimate the natural sterility of the mosquito population, as well as the overall egg hatch. Eggs collected were checked under a stereoscopic microscope (40 ×) to count the number of eggs per ovitrap and determine their status as *hatched, damaged, intact,* or *collapsed*. If identified as an egg (regardless of its status), it was summed to the total of that sample; otherwise, it was disregarded. After counting, the ovitrap paddles were individually submitted to the hatching process. Each paddle was placed in a sterile glass jar containing dechlorinated water at room temperature to stimulate egg hatching. The jars were maintained under controlled laboratory conditions (25 ± 2 °C, 12∶12 h light∶dark photoperiod), and the numbers of hatched larvae were recorded for each trap approximately 96 h after hatching commenced. It was considered the total number of larvae, the hatched larvae, and any egg classified as *hatched*.

#### Adult monitoring with BG-Sentinels

In 2019, a total of 37 and 30 BG-Sentinel traps (Biogents, Germany) were installed and georeferenced, in Brasilia Teimosa and Pina, respectively (Fig. 1B). The *Ae. aegypti* adult collection had a trap density of about 0.5 traps per hectare, and all traps used the BG-Lure attractant, according to the manufacturer’s instructions. All samples were sent to Moscamed Brasil, where all mosquitoes were identified to the genus category, but *Aedes* mosquitoes had their species and sex determined and recorded.

### Mosquito rearing, irradiation and shipment

The *Ae. aegypti* strain colony originated from eggs collected in six neighborhoods of Recife. In 2018, colonization began at the Biofábrica Moscamed Brasil mass-rearing unit in Juazeiro, Brazil, approximately 700 km from the target area (Fig. 1). The mass-rearing adult colony was maintained using standard climate-controlled conditions [27 ± 1 °C; 70 ± 10% relative humidity; and photoperiod of 12∶12 (light∶dark) hours] according to the protocol previously described [20]. The mass rearing description was provided in the Additional file 1.

**Fig. 2:**
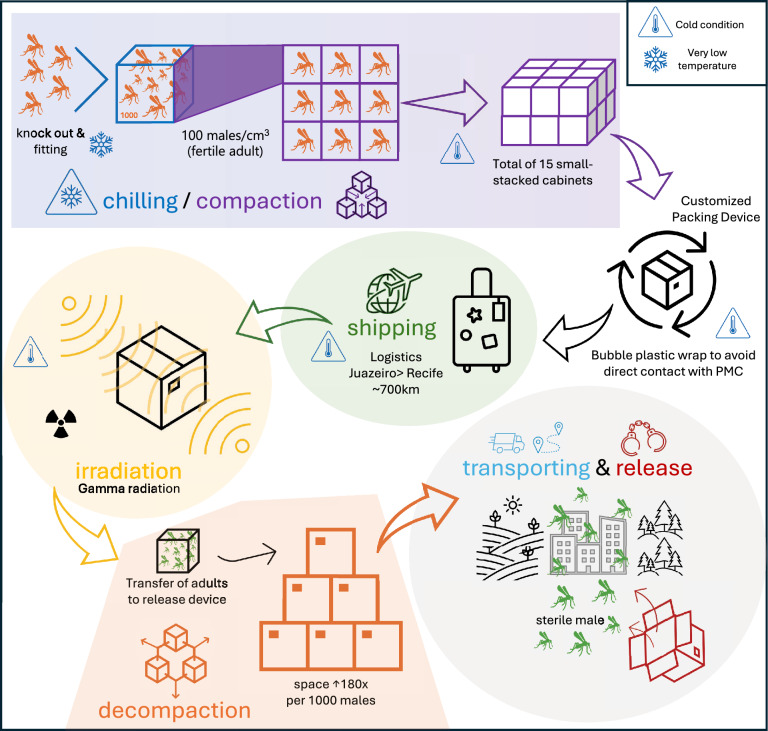
Step-by-step procedure from production to release of sterilized marked males. After the emergence of the adults, they are knocked out by chilling; later, males are compacted into cubes at a density of 100 males/cm^3^. Each group of nine cubes is stacked into a maximum of 15 layers, which are protected to be placed inside a final box for shipment over 700 km (from Juazeiro to Recife). Upon arrival, the material is sent directly to sterilization and decompaction. Then, the sterile males are marked with fluorescent powder and transported to the field for release

The *Ae. aegypti* males produced in Juazeiro were shipped to Recife, where the adults were sterilized and processed for release (Fig. 1A and 2). For compaction and transportation, we followed the methodology proposed by Gomez and colleagues in 2022 [15].where male mosquitoes were compacted to 100 adults/cm^3^ at 10 ± 1 °C. During the transport, temperature monitors collected data throughout the entire process. Upon arrival, samples were collected to assess the impact of transportation on them using the flight ability test. The Additional File 1 contains more details about the transporting male mosquitoes.

Following the phase conditional approach (PCA), we aimed a sterility dose more than 99%, and we performed a dose–response curve to determine the lowest dose required to achieve this sterility using X and gamma ray [21] and in pupae and adults (with different ages). Irradiation dosimetry details can be found in the Additional File 1, as well as detailed description for the dose–response in pupae and adults and the male irradiation routine for releases.

### Sterile male release and impact

The sterilized adult males were transported to the release site in Brasilia Teimosa (Fig. 1). Upon arrival, they had 72 to 96 h post-emergence and were immediately submitted for sterilization. During the early hours after sunrise, the ground releases took place by simply opening the release device lid and allowing the sterile, marked mosquitoes to fly out. Two routes were used simultaneously, with 10 release points for each, approximately 100 m apart. The first releases were from 26 October 2020 until 11 July 2021, with only one release per week. The second phase lasted from July 12, 2021, to April 7, 2022, with two releases per week. There were 20 release points with a varying number of sterile males at each location. Some points received more males according to the ovitrap data when the collection point was identified as a hotspot at that time.

#### Impact of handling and transporting males

Four stages were established to facilitate the collection of samples and execution of analysis as those steps (all described above) were taking place. Stage I (control) corresponds to the emerged males before handling, transportation, and irradiation. Stage II corresponds to males that were submitted to chilling, while stage III males were chilled, compacted, and then transported. The last stage, IV, involved chilling, compacting, transporting, irradiating, and marking the males. After stage IV, these males were ready for field releases. At this point, we evaluated the flight ability of these males, and upon their arrival and sterilization, we assessed the mortality after stage IV. The flight ability was assembled according to the guidelines and references available [22–24]. Briefly, for each stage (I to IV), 100 males were selected for this test, which was performed soon after they were collected. The flight ability lasted approximately 120 min, during which all escapers were counted from the initial total at the beginning of the test, and data were recorded.

#### Impact of sterile male releases

After the releases, approximately 5% of the release devices were randomly selected to assess mortality, which involved counting the number of insects inside the container (both non-fliers and deceased). The average percentage value was used to correct the initial total of sterile males; here, we present the average weekly percentage value used to adjust the initial data.

We determined the ETD, which is the average number of eggs per trap per day, by dividing the total number of eggs by the number of days the trap was in the field and then dividing this amount by the total number of positive traps remaining in the field. So the total number of eggs in this study is the sum of eggs collected in those installed traps, and not the total available eggs in the field. Meanwhile, the egg hatch rate was defined as the total number of hatched larvae divided by the total number of eggs collected across all traps, and data were reported weekly.

Induced sterility was calculated according to the formula: $IS= (1 - [Ho/Hn]) * 100$. Where *Ho* is the egg hatching observed in the treatment area, and *Hn* is the natural egg hatching [25]. The released males were marked with fluorescent powder, and after collection using the BG-Sentinel, the marked males could be distinguished from the wild males and females. The total number of marked and collected sterile males represents the recapture rate of mosquitoes from the total number of collected mosquitoes from that same species in the same trap/week. In addition to the adult collection, the sterile-to-wild males were calculated using the total number of sterile males divided by the total number of wild males captured at the same collection period (epidemiological week).

### Data analysis

The statistical analysis used R version 4.3.2—"Eye Holes"—(R Core Team, Vienna, Austria, 2023) and performed data visualization using RStudio: Integrated Development Environment for R (RStudio Team, 2024—“Ocean Storm” RStudio, Inc. (Posit Software, PBC), Boston, USA). Data distributions were adjusted to be binomial (negative or beta) or Poisson. Group comparisons were performed using generalized linear mixed models (GLMMs) with a significance level of 0.05, in which some variables were treated as fixed effects and others as random effects. For dose response curves, a generalized linear model (GLM) with the same type of distribution was used, considering the same alpha value. The model output is provided in Additional File 2 (Statistical Analysis Output), along with the R script.

In addition, a type of time series analysis was employed to estimate the impact of sterile male releases on the various measured parameters, including ETD, egg hatching, and the wild female population, using the R package “*CausalImpact*” [26]. This method employs a Bayesian structural time-series model to estimate the expected outcome in the absence of the intervention and compares it with the observed outcome after the intervention. By analyzing the difference between the predicted and actual outcomes, the “*CausalImpact*” function provides insights into the intervention's impact, as well as credible intervals to quantify uncertainty.

## Results

### Pupa and adult irradiation using X and gamma radiation

**Fig. 3:**
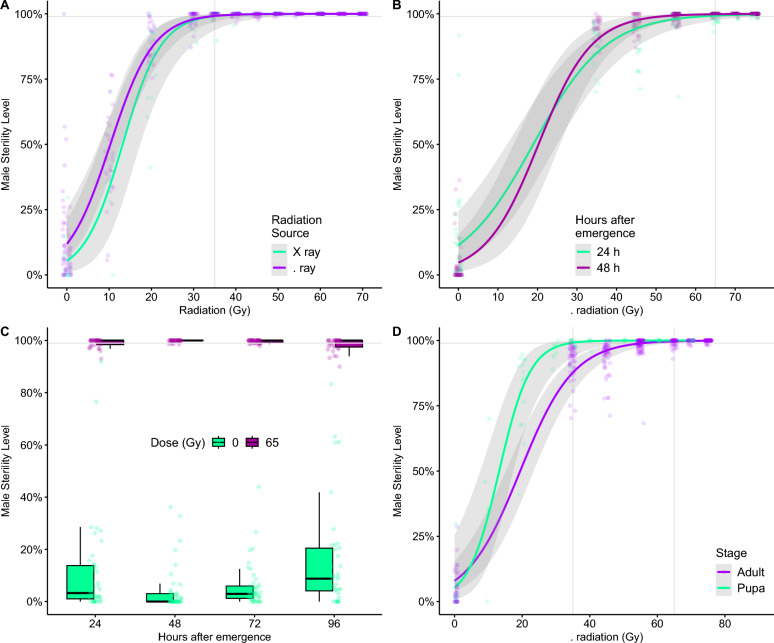
Dose–response curves for comparison of radiation between gamma and X radiation for pupae (**A**); dose–response curve gamma irradiation for adults 24 and 48 h after emergence (**B**); sterility level of different age groups of adults of 24, 48, 72, and 96 h after emergence irradiated with gamma source (**C**); the dose–response curve for the sterility rate of males pupae and adults irradiated with gamma source (**D**)

The gamma and X radiation to sterilize males of *Ae. aegypti* were compared using both pupal and adult stages. A series of dose–response curves, using doses ranging from 0 to 70 Gy, was performed to determine the minimal dose required to achieve 99% sterility in pupae. The results showed that the minimal dose for both sources at this life stage was identical at 35 Gy, Fig. 3 with no significant difference between the two types of source (GLM *F* = 0.079, *DF* = 1, 368; *P* = 0.78).

For adults of *Ae. aegypti*, the gamma irradiation doses were tested at 24 and 48 h after emergence of the males (Fig. 3B). Again, no statistical difference (GLM *z* = 1.25; *P* = 0.212) was found between the two age groups for the doses of 35, 45, 55, 65, and 75 Gy. According to this dose–response curve, the sterilizing dose for adults was 65 Gy. To explore further the effect of male age (after emergence), we also tested 72 and 96 h after emergence (Fig. 3C) at 65 Gy, resulting in no significant difference among the irradiated groups (GLM *F* = 0.072, *DF* = 1, 156; *P* = 0.789). The results suggest that adult males with 24–96 h after emergence can be grouped into the same radiation cycle (Fig. 3C). The 99% sterility level was only obtained when males were irradiated at 35 and 65 Gy for pupae and adults, respectively, (GLM *F* = 0.898, *DF* = 1, 94, and *P* = 0.346), showing that adult males require a higher dose (extra 30 Gy in our conditions) to reach the same sterility level of pupae irradiated with 35 Gy (Fig. 3D).

### Impact of handling and transporting males on their flight ability

**Fig. 4:**
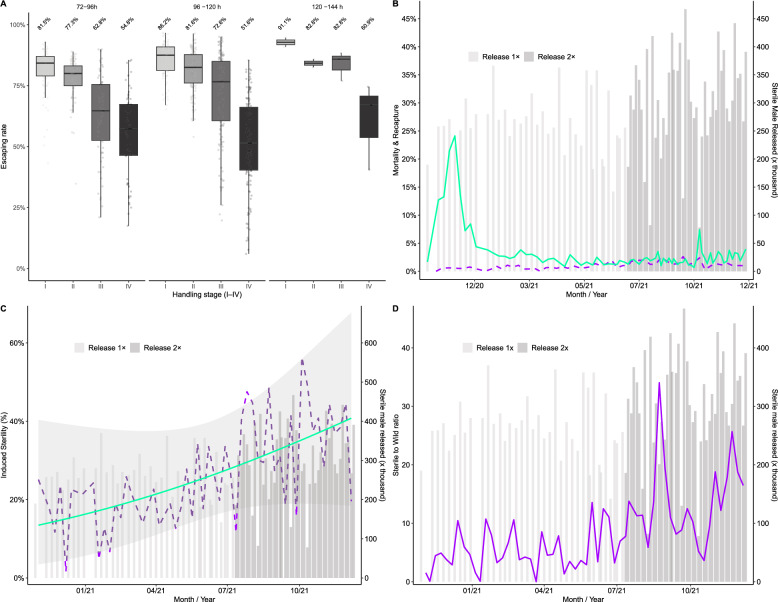
Panel for the impact of handling, transporting, and sterile release. **A** Escape rates of *Aedes aegypti* males, according to the treatment stage (I—control; II—chilled; III—stage II + compacted + transported; and IV—stage III + irradiated + marked), the flight ability test was conducted in three different groups of males (72–96, 96–120, and 120–144 h post-emergence). **B** Mortality and recapture rates after the field releases. **C** Induced sterility in Brasilia Teimosa during the release 1 × and 2 ×/week. **D** Sterile-to-wild ratio of males during the release 1 × and 2 ×/week

To produce sterile males and release them in the field, several steps were taken to ensure their success. However, at each step, the males are exposed to specific conditions that may increase their stress levels and reduce their overall fitness, resulting from the cumulative effects of the treatments they receive. The Fig. 2 summarizes all stages (I–IV), starting with the production of males, chilled knockout, compaction, packing, transportation, irradiation, decompaction, marking, and finally the field release. To assess the impact of cumulative treatment (stages I–IV) on males, we evaluated the flight ability of the males. The male escaping was analyzed as a proportion using a beta–binomial GLMM (logit) with stage (I–IV) and age (72–96 h, 96–120 h, 120–144 h) as fixed effects and a random intercept for the release batch (Fig. 4A). The model fit was good (*AIC* = 6251.0, *N* = 812 assays across 100 batches), with non-zero batch-to-batch variability (*SD*_batch_ = 0.393) and substantial extra-binomial dispersion (dispersion parameter = 19.8 – Additional File 2—Statistical Analysis Output). Within every age class, the model-estimated escape probability decreases stepwise from Stage I (minimal handling of males) to Stage IV (all handling steps upon release). Within every age class, the model-estimated escape probability decreases stepwise from Stage I (minimal handling of males) to Stage IV (all handling steps up to the release stage). The percentage of escaping males starts high (81–91% escape at stage I) and decreases by 27–35 percentage points by stage IV, depending on age. The early step (I and II) reduces the loss by only 4–5 points; the larger losses become apparent by stage III, especially stage IV. In words, each additional processing step reduces the odds of escape by 43% on average. Because this is an odds effect, the absolute drop in probability is modest at high baselines (e.g., 82–77% from I to II) and becomes larger as probabilities fall (e.g., 73–52% from III to IV in 96–120 h). Across all models, age neither changed the mean escape nor interacted with stage (all *P* > 0.1). Estimates in the 120–144 h class should be interpreted cautiously due to limited sample size (wide *CI*s), but the downtrend remains apparent. For fliers, the cumulative handling processing sequence reduces flight ability, with small early losses and a clear additional drop by stages III–IV. On an absolute scale, expect a 27–35 percentage-point lower escape rate at stage IV compared to stage I; on a relative (odds) scale, expect a 43% lower odds ratio per step on average (Fig. 4A).

### Impact of sterile male releases in the field population

After releasing sterile males, we observed that overall mortality was low and similar across the weekly release scenarios (1 × and 2 × per week). The mean weekly mortality rates were 4.8% (95% *CI* 1.5–14.0) in the 1 × release and 4.5% (95% *CI* 1.4–13.5) in the 2 × release (Fig. 4B). A binomial GLMM with random effects for year and week revealed no significant difference between phases (estimate = –0.070 ± 0.117 on the logit scale, *Z* = −0.60, *P* = 0.55; odds ratio = 1.07, 95% *CI* overlapping 1).

After releases, the recapture rates of sterile males in BG traps were higher during 2 × release than during 1 × release. Weekly mean recapture was 0.67% (*SE* = 0.10) in 1 × release vs. 1.46% (*SE* = 0.09) in 2 × release. The Gaussian GLMM indicated a strong phase effect (estimate = 0.0083 ± 0.0019, *Z* = 4.40, *P* < 0.001), while the slope of mortality (% dead before release) on recapture was negative but non-significant (estimate = −0.0006 ± 0.0007, *P* = 0.41). This suggests that batch-to-batch mortality fluctuations had limited predictive power, whereas doubling the release frequency clearly improved sterile male field presence.

**Fig. 5:**
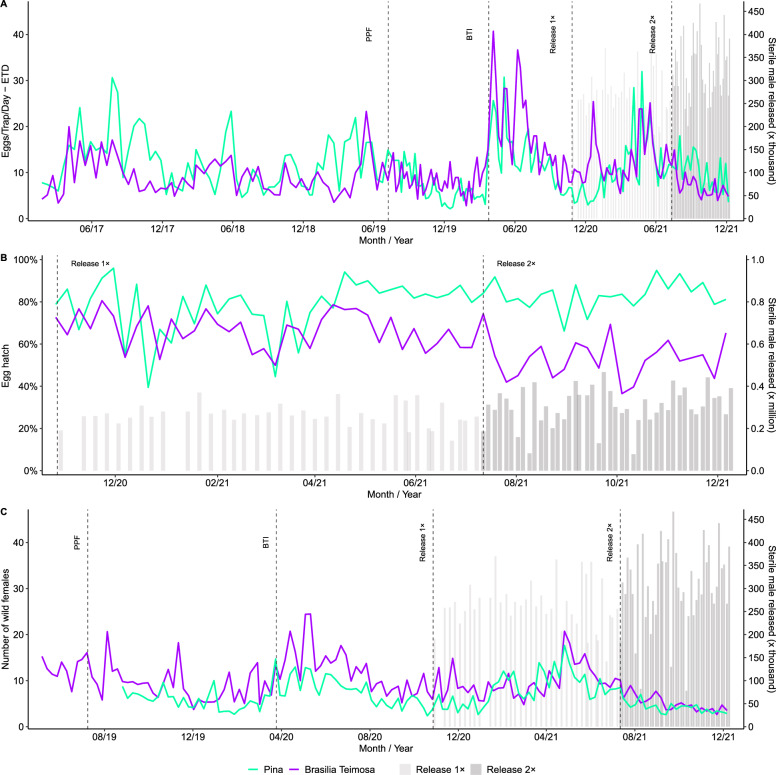
Field data from Pina (non-intervention area in green) and Brasilia Teimosa (intervention area in purple), showing the baseline phase period followed by chemical and sterile male release interventions (*PPF* pyriproxyfen, Bti, releases 1 × and 2 ×/week). In which **A** shows the number of eggs/trap per day (ETD), **B** the egg hatching, and **C** the number of wild females captured, with the amounts of sterile males shown in grey bars. *Bti Bacillus thuringiensis israelensis*

The number of eggs per trap per day (ETD - Fig. 5) was significantly affected by intervention phases and their interaction with location (Γ-log, *σ*^2^ = 0.161, *n* = 352). During the baseline, ETD was already lower in Brasília Teimosa (emmean 9.16 ETD, 95% *CI* 8.03–10.28) than in Pina (12.2 ETD, 95% *CI* 10.8–13.7), with a highly significant difference (GLMM, estimate = −0.29, *SE* = 0.015, *P* < 0.0001). Before releases started, it was expected that applying Bti would begin to reduce the mosquito population. With the larvicide interventions, PPF showed slightly higher ETD in Brasília, 7.98 vs. 7.4 ETD in Pina (estimate =  + 0.07, *SE* = 0.027, *P* = 0.0078). During the Bti phase, this divergence became stronger, with Brasília reaching 25.75 ETD versus 18.7 ETD in Pina (estimate =  + 0.32, *SE* = 0.026, *P* < 0.0001). Under sterile male releases, contrasting patterns were observed (Table 1). With one weekly release (1 × release/week), ETD in Brasília remained higher than in the control 12.0 vs. 9.9 ETD (estimate =  + 0.18, *SE* = 0.022, *P* < 0.0001), suggesting limited suppression. In contrast, with two releases per week (SIT 2 ×), Brasília exhibited a marked reduction (6.3 ETD) compared to Pina (9.0 ETD; estimate = −0.35, *SE* = 0.029, *P* < 0.0001). These results demonstrate that SIT was only effective in reducing oviposition when sterile males were released twice weekly, resulting in 35% fewer eggs per trap per day compared to the control (Fig. 5).

**Table 1:** Primary outcomes in Brasília Teimosa during the different phases before interventions—Baseline, during interventions PPF – Pyriproxyfen, Bti, and the sterile male release phases (1 × and 2 × release/week)

Period	ETD	H (%)	IS (%)	R (%)	ST:WT
Baseline	9.16 (8.03–10.28)	N/A	N/A	N/A	N/A
PPF	7.98 (6.99–8.96)	80.5 (78.4–82.6)	N/A	N/A	N/A
Bti	25.75 (22.44–29.06)	39.4 (36.4–42.4)	N/A	N/A	N/A
1 × release/week	12.0 (10.44–13.55)	67.7 (64.9–70.5)	18.9 (15.7–22.0)	0.67 (0.57–0.77)	3.10 (± 2.87)
2 × releases/week	6.30 (5.45–7.15)	55.4 (52.1–58.7)	32.2 (27.6–36.8)	1.46 (1.37–1.55)	17.53 (± 17.69)

Values are reported as the mean (95% *CI*)

*ETD* Eggs/trap per day, H(%) as egg hatch rate in percentages, IS (%) as the percentage of induced sterility in the wild females, R (%) as the recapture percentage from the total released, and the ST:WT as the sterile-to-wild male proportion found in the BG-Sentinel traps. N/A not applicable, *Bti Bacillus thuringiensis israelensis. *The sterile-to-wild male ratio is reported as the geometric mean with the standard deviation

The egg hatching was assessed using a beta regression model with a logit link (Fig. 5), which revealed significant differences between locations (Brasilia Teimosa and Pina) and intervention phases—insecticide application and releases of sterile males (GLMM, *n* = 214; σ^2^ = 22.7; *AIC* = −8660.9). During the PPF phase, hatching was higher in Pina (85.7%, 95% *CI* 83.8–87.4%) compared to Brasília Teimosa (80.5%, 95% *CI* 78.4–82.6%), with a significant difference between sites (GLMM, estimate = −0.372 ± 0.039, *P* < 0.0001). In contrast, during the Bti phase, Brasília exhibited higher hatching (39.4%, 95% *CI* 36.4–42.4%) than Pina (33.7%, 95% *CI* 30.8–36.7%; estimate = 0.246 ± 0.028, *P* < 0.0001). For the sterile male phases, Pina consistently maintained higher levels of egg hatching (fertility). In the 1 × release, mean hatching reached 80.4% (95% *CI* 78.2–82.4%) versus 67.7% in Brasília (95% *CI* 64.9–70.5%; estimate = −0.666 ± 0.026, *P* < 0.0001). This divergence widened further during 2 × release (Table 1), when hatching in Pina remained at 83.8% (95% *CI* 81.7–85.6%), while Brasília dropped to 55.4% (95% *CI* 52.1–58.7%; estimate = −1.423 ± 0.036, *P* < 0.0001).

The induced sterility was evaluated using a beta regression model (logit link - Fig. 4C), and it showed strong intervention phase effects (GLMM, *n* = 104; σ^2^ = 17.1; *AIC* = −3698.7—Table 1). During 1× release, induced sterility was low in Brasilia Teimosa, averaging 18.9% (95% *CI* 15.9–22.2%). Under 2 × release, induced sterility increased markedly, reaching 32.2% in Brasília (95% *CI* 27.8–36.9%). Thus, sterile male releases, approximately twice per week, induced sterility that was approximately doubled compared to 1 × release/week.

The male sterile to wild ratio was analyzed using a Gaussian, log-transformed ratio (GLMM, *n* = 11,802; σ^2^ = 0.86; *AIC* = 31,875), revealing strong effects of release frequency (Fig. 4D and Table 1). During 1× release, the sterile:wild ratio was lower in Brasília Teimosa (geometric mean 3.10 ± 2.87 *SD* on the back-transformed scale) than under 2 × release, where ratios increased further in Brasília (geometric mean 17.53 ± 17.69 *SD* on the back-transformed scale). Thus, sterile male releases twice per week markedly increased the sterile:wild male ratio in the release area, surpassing the levels observed under 1 × release/week (Table 1). As expected, no sterile males were detected in the control area (Pina, 0 ± 0).

### Causal impact analysis

**Fig. 6:**
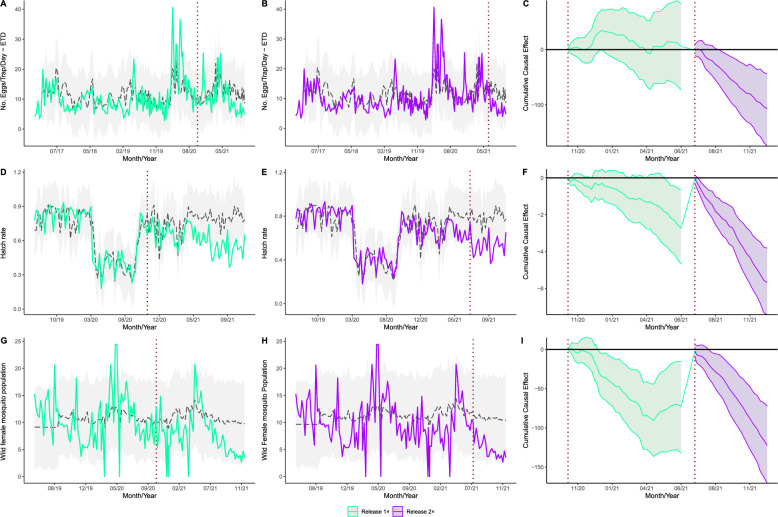
Causal impact analysis of releases performed in Brasilia Teimosa. Model analysis of the number of eggs/trap per day with males released once a week (**A**), and twice a week (**B**), with its cumulative effect (**C**). Model analysis of the hatch rate of males released once a week (**D**), and twice a week (**E**), with its cumulative effect (**F**). Model analysis of the number of wild females during the release of males once a week (**G**), and twice a week (**H**), with its cumulative effect (**I**). The dashed line represents the model expected values, while the colored continuous line represents the observed data (in **A**, **B**, **D**, **E**, **G**, and **H**)

To determine whether the release of sterile-marked male mosquitoes affected the wild population, an interrupted time series analysis was performed using the “CausalImpact” function, which assesses the causal effect of an intervention on a time series dataset. This function enables the estimation of the causal impact of a predefined intervention on a time series, taking into account the baseline data. The average number of ETD was approximately 12.45 during the intervention period, in which sterile males were released once a week. Without intervention, we would have expected an average response of 12.62. The once-a-week SIT intervention estimated causal effect thus showed an impact of 0% (with a 95% interval of −16%, 21%—Fig. 6A). We also performed this analysis for the hatch rate, estimating an average of 0.67 during phase one of the intervention period (one release/week). In contrast, the expected response, according to the model, would have been 0.76. The 11% decrease (with a 95% interval of −18% to −3%) caused by releasing sterile males once a week was statistically significant, with a Bayesian one-sided tail-area probability of *P* = 0.007 (Fig. 6D). During phase one of the intervention, the mean number of females per week, as estimated by the causal impact analysis, was 9.18. Without the SIT intervention, it would be expected to have 11.28 females/week, representing an 18% reduction (with a 95% interval of −30% to −4%), again statistically significant with a Bayesian one-sided tail-area probability of *P* = 0.009 (Fig. 6G).

The average number of eggs per trap per day (ETD) was approximately 7.12 during the intervention period, in which sterile males were released twice a week. Without any intervention, we would have expected an average response of 11.99. The twice-a-week SIT intervention resulted in an estimated decrease of 39% (with a 95% confidence interval of −53%, −19%), which was statistically significant with a Bayesian one-sided tail-area probability of *P* = 0.002 (Fig. 6B). The hatch rate was estimated to be 0.53, while the expected response without intervention was 0.79. The intervention of releasing sterile males twice a week resulted in a 33% decrease (with a 95% confidence interval of −39%, −25%), which was statistically significant with a Bayesian one-sided tail-area probability of *P* = 0.001 (Fig. 6E). Additionally, the mean number of females per week estimated by the causal impact analysis was 5.23. However, without the SIT intervention, it would be expected to reach 10.84 females per week, representing a 51% reduction (with a 95% interval of −60% to −39%), which is statistically significant with a Bayesian one-sided tail-area probability of *P* = 0.001 (Fig. 6H).

Overall, the cumulative causal impact model illustrates the long-term effect of the SIT intervention. It calculates the net change caused by the treatment throughout the post-intervention period and displays the confidence interval. The results indicate that the intervention decreases all mosquito parameters under study, with a more substantial decline in the mosquito population during the second phase of the intervention (Fig. 6C, F, and I).

## Discussion

This Recife pilot trial offers key insights into the operational feasibility and biological outcomes of SIT in a densely populated tropical urban environment. The study showed that it is possible to suppress a single *Ae. aegypti* population, but this requires doubling the release frequency, emphasizing the need for consistent sterile male releases in the field [27, 28].

Our findings indicate no difference between gamma and X ray irradiation in producing sterile males, with pupae requiring 35 Gy and adults 65 Gy to achieve > 99% sterility. These results are in agreement with previous studies, which show equivalence between radiation sources [25, 29, 30], and they reinforce the pupal sensitivity to irradiation compared to adults [31–33]. The ability to irradiate adults up to 96 h post-emergence without loss of sterility provides valuable logistical flexibility, as mixed-age cohorts can be processed together, reducing operational constraints and simultaneously increasing the number of males that can be used [34, 35]. This is particularly advantageous compared with pupal irradiation, which requires precise timing and discarding of uncertain-aged individuals [36–38].

Flight ability assays revealed that each additional handling stage (chilling, compaction, transport, irradiation, and marking) reduced escape probability, resulting in cumulative losses of ~ 27–35 percentage points between untreated (stage I) and fully processed, or pre-release males (stage IV). These findings confirm that long-distance transport imposes measurable quality costs [15, 16, 39], consistent with reports from other SIT programs facing similar logistics challenges [40, 41]. While adult mortality before release remained relatively low (approximately 5%), the apparent reduced field competitiveness, which translates from the lower flight ability, underscores the need for either localized production or improved handling and transportation conditions [42, 43]. A preliminary study in Fernando de Noronha has highlighted the importance of evaluating local mosquito susceptibility and physiological responses to irradiation before operational releases [44, 45].

Entomological monitoring revealed that 1 × release had a minimal suppressive effect. In fact, ETD values during 1 × release remained higher in the intervention area than in the control, suggesting that a single weekly release was insufficient to offset the reproductive capacity of the wild population. Conversely, the 2 × releases consistently reduced ETD (–39%), hatch rate (−33%), and female abundance (−51%). These results align with the expectation that sterile:wild ratios must surpass threshold levels, in our case 17:1, to initiate the population suppression phase, which is recommended to range from 10 to 100 sterile males for each wild male [46, 47]. The observed induced sterility (approximately 32%) in the 2 × release was almost twice that of the 1 × release, reinforcing that frequency is a decisive operational parameter.

While raw indices (ETD, hatch rate, female abundance) showed variability and sometimes non-significant differences between sites, time-series modeling (CausalImpact [26, 48]) provided robust evidence of intervention effects. This underscores the need to complement traditional entomological monitoring with advanced statistical approaches for evaluating SIT outcomes [42, 49, 50].

Several field trials worldwide provide valuable context for interpreting our findings. In Cuba, a large-scale SIT trial using irradiated *Ae. aegypti* males achieved significant suppression of local populations in Havana, demonstrating that sterile releases can be effective in dense urban environments when production is local and logistics are simplified [11]. Similarly, in Captiva Island (Florida, USA), SIT achieved near elimination of *Ae. aegypti* over 2 years, facilitated by the insular and controlled setting [27]. In Europe, pilot releases of *Ae. albopictus* in Italy and Spain reported substantial reductions in egg hatch rates and female abundance, particularly when SIT was integrated into broader vector management programs [9, 28]. More recently, “boosted” SIT trials in Spain and La Réunion achieved drastic population declines (> 90%) when release intensity was maximized, confirming the importance of frequent and large-scale releases [51]. Taken together, these experiences show that the efficacy of SIT depends strongly on release frequency, local production capacity, and ecological context. Our Recife trial aligns with these global findings: twice-weekly releases led to substantial suppression. In contrast, once-weekly releases were insufficient, underscoring both the promise of SIT in complex tropical cities and the operational need to optimize logistics and male quality.

**Implications for scaling SIT:** The Recife trial demonstrates that SIT can function effectively in highly urbanized tropical contexts, provided that release frequency is optimized and fitness is maintained. However, transport-related fitness losses represent a bottleneck that may limit scalability [15]. While our results suggest that release frequency influenced suppression outcomes, we acknowledge that the sequential (rather than simultaneous) design of the 1 × and 2 × release phases limits direct comparability, despite a few environmental changes occurring during the study. Operational constraints in male production and in the field dictated this design; however, the use of time-series modeling mitigates, to some extent, the potential confounding effects of temporal variation [27, 52]. Furthermore, we did not perform direct laboratory or semi-field competitiveness assays for sterile males. Instead, flight ability and recapture rates were used as proxies for field performance [22, 43, 53]. Subsequent trials should incorporate standardized competitiveness tests to complement operational indicators. Future programs should consider decentralized production units near release sites or invest in improved packaging and chilling protocols [14, 54–56]. Operational lessons from Recife—including irradiation flexibility, impact monitoring, and release frequency optimization—are directly applicable to other endemic regions seeking to integrate SIT into broader vector management strategies.

## Conclusions

This pilot trial in Recife demonstrates that the sterile insect technique (SIT) can be implemented even in complex tropical urban settings. Our results show that only by doing releases twice a week, consistently, were we able to observe the *Ae. aegypti* population drops, while the once-per-week release could not be shown. This suppression level was determined by the reduced egg density, hatch rates, and adult female abundance found in both areas. At the same time, handling and transport negatively impacted the sterile males, underscoring logistics as a critical operational bottleneck. These findings align Recife with international SIT experiences, reinforcing that frequency and logistics are decisive factors in initiating suppression, along with male fitness. Future programs will require local production, rigorous fitness control, and integration into broader vector management strategies to maximize effectiveness and sustainability. In the end, the lesson from Recife is clear: with the exemplary commitment, SIT is not only feasible—it is powerful enough to bend the curve of mosquito-borne disease transmission.

## Supplementary Information

Additional file 1.

Additional file 2.

## Data Availability

The datasets used and/or analysed during the current study are available from the corresponding author on reasonable request.

## Abbreviations

SIT: Sterile insect technique
RH: Relative humidity
H(%): Hatch rate
R(%): Recapture rate
IS: Induced sterility
ETD: Eggs/trap per day
PPF: Pyriproxyfen
Bti: *Bacillus thuringiensis israelensis*
STWT: Sterile type to wild type male ratio
GLMM: Generalized linear mixed model
GLM: Generalized linear model
BRT or B. Teimosa: Brasilia Teimosa
PIN: Pina
*SE*: Standard error
*CI*: Confidence interval
BG: Biogents, Germany
