# Supplementary material for: Field implementation of the sterile insect technique against Aedes aegypti in Recife, Brazil: operational challenges and impact of release frequency on vector dynamics
Source: Infect Dis Poverty. 2026 Jan 29;15:16. doi: 10.1186/s40249-025-01393-7 (PMC12853722; doi:10.1186/s40249-025-01393-7)
Supplement: Supplementary file 1 — Additional file 1. [file 40249_2025_1393_MOESM1_ESM.docx]

# Additional file 1

### Mass rearing

Females, at a ratio of 3∶1 (female to male), were used for egg production by allocating the desired number of insects into plastic containers and placing them into PVC cages (30 cm in height and 30 cm in diameter). After emergence, the adults had constant access to a 10% sucrose solution. Once a week, females were offered a blood meal using an artificial system with goat blood (0.06 μl/female/gonotrophic cycle). Three days after the blood feeding, oviposition containers containing 200 ml of water (total volume of 500 ml; 8 cm height and 9.5 cm diameter) were placed in cages for female oviposition (oviposition paper strips: 41 cm wide and 8 cm high). The collected eggs were then transferred to drying trays, removed from the oviposition strips using a brush, weighed, and stored in plastic cups (50 ml), kept in a room under insectary-controlled conditions for future hatching. The larval rearing process began with egg hatching using previously prepared boiled water jars (sealed with 400 ml of boiled water) and adding 1 g of eggs/jar for two hours. The larva rearing tray (9.7 cm in height × 30.3 width cm × 51 cm length) received the hatched larvae at defined density (2.25 larvae/ml; 6,750 larvae/tray in total) and the larval diet (Sera Vipan Premium®, Sera - Germany) according to the feeding regime for the next seven days until development of pupae and the beginning of sex sorting, to separate males and females, using an adapted glass plate separator [1]. Using a calibrated spoon, we placed 1000 male pupae into emergence containers (1.8 L, 14 cm height, and 13 cm diameter) with approximately 150 ml of water. Samples were collected to serve as the control of the subsequent steps until release. At one point, these pupae were subjected to radiation (gamma and X) and then returned to their pots for adult emergence. Alternatively, for adult irradiation, the pupae remained in these pots until emergence. They were transported and irradiated, initially for age comparison purposes, and later as part of the continuous release phase aimed at suppressing the field population.

#### Transporting device

Commercial flights transported shipment containers (Styrofoam boxes 16 × 19.3 × 22 cm - length, width, and height). For shipping, a custom-made packaging device of polymethyl methacrylate (PMMA - 10.4 × 10.4 × 22.9 cm) was built, which accommodated 15 small-stacked cabinets (7.1 × 7.1 × 2.4 cm - length, width, and height) containing nine cubes (2.2 × 2.2 × 2.2 cm, 10.6 cm^3^ volume), each one, totaling 135 cubes. Each cube was filled with 1000 males at a density of 100 adult males/cm^3^ (approximately 135,000 mosquito males/shipment device). Three units of a phase change material (PCMs - ClimSelTM C7) were placed around the exterior of the shipment container to maintain the desirable transport temperature (10 ± 1 °C), which kept the mosquitoes knocked out and immobilized during the shipping process. However, to prevent direct contact between the PCMs and the packaging device, the latter was covered with plastic bubble wrap and then centrally positioned within a polyfoam box. The entire handling process was carried out inside a walk-in cold chamber (230 cm *×* 230 cm—Genfrio Refrigeration Commerce and Service Ltd., Recife, Brazil) with a temperature range of *±* 1 °C, at a temperature of approximately 5 ± 1 °C, and took approximately 45 minutes.

### Irradiation

#### X and gamma irradiators and dosimetry

The X irradiator was a RadSource RS 2400 (Rad Source Technologies Inc., Suwanee, USA) operated at 125 kV Voltage, 18 mA current, and a dose-energy ratio of 0.07452 kGy/kW^-1^h^-1^, while the gamma irradiator was a ^60^Co Gammacell-220 (MDS Nordion, Ottawa, Canada) with a dose rate of 1.59 kGy/h and a dose uniformity ratio (DUR) of 1.84 at the beginning of the experiments. Dosimetry was performed using alanine dosimeters (Aérial, France). The dosimeters were encapsulated in plastic badges and placed near the samples to verify the dose absorbed by the biological material during irradiation with the nominal doses. Alanine-EPR measurements were conducted using a Bruker Magnettech ESR 5000 spectrometer with a standard cylindrical resonator cavity in the X-band. The microwave power was 10 mW, with a modulation amplitude of 0.7 mT and a frequency of 100 kHz. The EPR response was determined by the peak-to-peak amplitude of the most intense peak, proportional to the concentration of free radicals in the alanine dosimeter.

#### Pupae irradiation dose-curve response

At the pupae stage, dose-response curves were defined for gamma and X radiation in *Ae. aegypti* males. The first step after pupae sorting was to place 100 male pupae in the central portion of the canister (one group for gamma and another for X ray) for each tested dose, which ranged from 0 (control) to 70 Gy. After their emergence, 75 irradiated males mated with 75 fertile virgin females. The mated females received a blood meal for egg development, and three days later, they were separated individually and offered an oviposition substrate. The egg paper was dried, and their eggs were counted and hatched as described previously, to determine the fecundity and fertility (resulting in the male sterility level).

#### Adult irradiation dose-curve response

After male adult emergence, 225 male adults were placed in the central middle of the canister for each tested dose, ranging from 0 (control) to 70 Gy using gamma rays. After exposure, 75 irradiated males were mated with 75 fertile virgin females. The mated females received a blood meal for egg development, and three days later, they were separated individually and offered an oviposition substrate. The egg paper was dried, and their eggs were counted and hatched as described previously, to determine the fecundity and fertility (resulting in the male sterility level).

### Male adult irradiation routine

Upon arrival in Recife, the Department of Nuclear Energy (DEN) of the Federal University of Pernambuco received the adults for sterilization. It utilized the previously mentioned Gammacell-220 irradiator, as described above. For sterilization, the small-stacked cabinets containing the cubes with the mosquitoes were transferred to a holding tower (17.7 cm in height x 7.7 cm in length) to keep all the stacked cabinets secure inside the irradiation chamber. This holding tower was built to ensure the recommended DUR for mosquito sterilization (i.e., DUR < 1.3) and, therefore, a minimal variation in the sterilizing dose within the irradiation volume. About 27,000 mosquitoes were sterilized per cycle (cycle duration ranged between 164 and 187 seconds based on the 60Co source’s activity and machine operation). To perform the dosimetry of sterile males for field release, thermoluminescent dosimeters (TL) of MTS-N (LiF: Mg, Ti), produced by the Institute of Nuclear Physics (INP) in Krakow, Poland, were used. They were encapsulated in a plastic badge and positioned on top of the cabinets. The readings were taken using a Harshaw 3500 TL reader with a heating rate of 15 °C/s and a maximum temperature of 300 °C. Following irradiation, males were kept compacted and knocked out during transportation to the field insectary, where they were placed in plastic pots (release device - 1.8 L; 1000 males/pot) and marked using fluorescent dye powder (0.025 g powder/1000 insects), at this point samples were collected to assess the impact of irradiation and marking, and also the determination of the mortality after all steps from mass rearing to field releases.

## Community awareness

Considering the project's goal to introduce the sterile insect technique (SIT) and validate its use in *Aedes aegypti* control strategies, field activities commenced with the establishment of baseline data and the colonization of the strain with a local genetic background, alongside Public Awareness actions. These actions aimed to ensure the dissemination and understanding of the project, involving public authorities and the community. The actions were planned to reach as many inhabitants of the communities as possible, considering the cultural and social context of the Brasília Teimosa community (treated area). Visits were made to residents in the pilot areas to inform them about the project's objectives and the functioning of Sterile Insect Techniques. These visits were conducted by Moscamed professionals and Health Agents from the Recife City Hall. The Brasília Teimosa Residents' Council served as the focal point for meetings between local leaders and the community, as well as between Moscamed technicians and epidemiological surveillance system technicians. The information/education campaigns aimed to reach two major groups of people: i) individuals in the formal education program, including students and teachers, and ii) individuals targeted by informal education actions, mainly family leaders and workers. The project was presented in schools and community centers in the pilot areas to ensure different communities gained knowledge on topics such as insect biology, integrated vector control strategies, and autocidal control. A Media Plan was developed in advance of mosquito release operations, with jingles/spots produced beforehand and disseminated via sound cars and/or radios both before and throughout the mosquito release activities. The primary communication vehicle was community media instruments, notably bicycles equipped with sound systems, known locally as "Anuncicletas" (bicycles used for announcements). Additionally, interviews were conducted on television and radio, informative pamphlets were distributed (providing information on the integrated control strategy), and the action and schedule were continuously disseminated on social networks and specific websites.

## References

1. Fay RW, Morlan HB. A mechanical device for separating the developmental stages, sexes and species of mosquitoes. Mosq News. 1959;19:144–7.
