# Supplementary material for: Field implementation of the sterile insect technique against Aedes aegypti in Recife, Brazil: operational challenges and impact of release frequency on vector dynamics
Source: Infect Dis Poverty. 2026 Jan 29;15:16. doi: 10.1186/s40249-025-01393-7 (PMC12853722; doi:10.1186/s40249-025-01393-7)
Supplement: Supplementary file 2 — Additional file 2. [file 40249_2025_1393_MOESM2_ESM.docx]

# Additional File 2

# Statistical Analysis Output

## Irradiation Pupa Dose-response curve X

glm(formula = hatching ~ dose_fac, family = binomial(link = "logit"),

data = radiation %>% filter(source == "x-ray", stage == "pupa",

n_eggs > 10))

Coefficients:

Estimate Std. Error z value Pr(>|z|)

(Intercept) -8.4687 772.2669 -0.011 0.991

dose_fac.L -25.5605 3502.1324 -0.007 0.994

dose_fac.Q -5.8356 2838.4715 -0.002 0.998

dose_fac.C -2.6962 2853.7207 -0.001 0.999

dose_fac^4 4.1691 3217.7911 0.001 0.999

dose_fac^5 5.9096 2938.4953 0.002 0.998

dose_fac^6 0.9667 2798.3198 0.000 1.000

dose_fac^7 -2.4692 3074.4972 -0.001 0.999

dose_fac^8 -2.9063 2890.8950 -0.001 0.999

dose_fac^9 -2.9018 2075.7158 -0.001 0.999

dose_fac^10 -2.8688 1088.4842 -0.003 0.998

dose_fac^11 -0.5670 368.7569 -0.002 0.999

(Dispersion parameter for binomial family taken to be 1)

Null deviance: 271.716 on 387 degrees of freedom

Residual deviance: 19.142 on 376 degrees of freedom

AIC: 101.69 Number of Fisher Scoring iterations: 20

## Irradiation Pupa Dose-response curve Gamma

glm(formula = hatching ~ dose_fac, family = binomial(link = "logit"),

data = radiation %>% filter(source == "gamma", stage == "pupa",

n_eggs > 10))

Coefficients:

Estimate Std. Error z value Pr(>|z|)

(Intercept) -8.448 1677.285 -0.005 0.996

dose_fac.L -26.866 6175.565 -0.004 0.997

dose_fac.Q -4.360 5446.672 -0.001 0.999

dose_fac.C 2.940 5977.886 0.000 1.000

dose_fac^4 5.788 4991.796 0.001 0.999

dose_fac^5 2.377 4294.404 0.001 1.000

dose_fac^6 -3.262 5346.515 -0.001 1.000

dose_fac^7 -4.866 4650.102 -0.001 0.999

dose_fac^8 -2.865 2334.315 -0.001 0.999

(Dispersion parameter for binomial family taken to be 1)

Null deviance: 80.5592 on 114 degrees of freedom

Residual deviance: 6.1019 on 106 degrees of freedom

AIC: 32.549 Number of Fisher Scoring iterations: 20

## Irradiation Pupa Gamma vs. X

Family: binomial ( logit )

Formula: hatching ~ source

Data: radiation %>% filter(stage == "pupa", n_eggs > 10)

AIC BIC logLik -2*log(L) df.resid

479.4 487.8 -237.7 475.4 501

Conditional model:

Estimate Std. Error z value Pr(>|z|)

(Intercept) -1.5720 0.2471 -6.361 2.01e-10 ***

sourcex-ray 0.0787 0.2798 0.281 0.778

---Signif. codes: 0 ‘***’ 0.001 ‘**’ 0.01 ‘*’ 0.05 ‘.’ 0.1 ‘ ’ 1

## Irradiation Adult control

glm(formula = hatching ~ age_fac, family = binomial(link = "logit"),

data = adult_age %>% filter(dose_fac == "0"))

Coefficients:

Estimate Std. Error z value Pr(>|z|)

(Intercept) 2.55083 0.28154 9.060 <2e-16 ***

age_fac.L -0.34088 0.52373 -0.651 0.5151

age_fac.Q -1.26620 0.59799 -2.117 0.0342 *

age_fac.C 0.01687 0.65850 0.026 0.9796

age_fac^4 -0.03732 0.72099 -0.052 0.9587

---Signif. codes: 0 ‘***’ 0.001 ‘**’ 0.01 ‘*’ 0.05 ‘.’ 0.1 ‘ ’ 1

## Irradiation Adult 24 - 96 h

glm(formula = hatching ~ age_fac * dose_fac, family = binomial(link = "logit"),

data = radiation %>% filter(stage == "adult", n_eggs > 10,

round == "A"))

Coefficients:

Estimate Std. Error z value Pr(>|z|)

(Intercept) -3.80854 1.00758 -3.780 0.000157 ***

age_fac.L -0.65999 1.42493 -0.463 0.643239

dose_fac.L -7.93029 3.17343 -2.499 0.012456 *

dose_fac.Q 1.88740 2.62388 0.719 0.471946

dose_fac.C -1.22981 2.85873 -0.430 0.667055

dose_fac^4 1.32460 2.93700 0.451 0.651986

dose_fac^5 -0.85311 2.50479 -0.341 0.733411

dose_fac^6 0.23716 1.61601 0.147 0.883322

age_fac.L:dose_fac.L -1.50835 4.48791 -0.336 0.736801

age_fac.L:dose_fac.Q 0.70580 3.71072 0.190 0.849149

age_fac.L:dose_fac.C 0.05041 4.04285 0.012 0.990051

age_fac.L:dose_fac^4 1.01076 4.15354 0.243 0.807736

age_fac.L:dose_fac^5 0.01383 3.54231 0.004 0.996886

age_fac.L:dose_fac^6 -0.29645 2.28538 -0.130 0.896791

---Signif. codes: 0 ‘***’ 0.001 ‘**’ 0.01 ‘*’ 0.05 ‘.’ 0.1 ‘ ’ 1

(Dispersion parameter for binomial family taken to be 1)

Null deviance: 399.668 on 536 degrees of freedom

Residual deviance: 26.259 on 523 degrees of freedom

AIC: 68.722 Number of Fisher Scoring iterations: 10

## Irradiation Pupa vs Adult

glm(formula = hatching ~ stage, family = binomial(link = "logit"),

data = pupa_rad)

Coefficients:

Estimate Std. Error z value Pr(>|z|)

(Intercept) -5.3167 1.6453 -3.232 0.00123 **

stagepupa 0.5519 2.9449 0.187 0.85134

---Signif. codes: 0 ‘***’ 0.001 ‘**’ 0.01 ‘*’ 0.05 ‘.’ 0.1 ‘ ’ 1

(Dispersion parameter for binomial family taken to be 1)

Null deviance: 2.0056 on 95 degrees of freedom

Residual deviance: 1.9730 on 94 degrees of freedom

AIC: 5.0838 Number of Fisher Scoring iterations: 8

## Flight ability

Family: betabinomial ( logit )

Formula: cbind(escaped, not_escaped) ~ stage * age + (1 | release_batch)

Data: flight_f

AIC BIC logLik -2*log(L) df.resid

6251.0 6316.8 -3111.5 6223.0 798

Random effects:

Conditional model:

Groups Name Variance Std.Dev.

release_batch (Intercept) 0.1542 0.3926

Number of obs: 812, groups: release_batch, 100

Dispersion parameter for betabinomial family (): 19.8

Conditional model:

Estimate Std. Error z value Pr(>|z|)

(Intercept) 1.14187 0.15090 7.567 3.82e-14 ***

stage.L -1.19675 0.14361 -8.333 < 2e-16 ***

stage.Q -0.16721 0.13901 -1.203 0.229

stage.C -0.09631 0.13457 -0.716 0.474

age.L 0.44058 0.31738 1.388 0.165

age.Q 0.06265 0.18869 0.332 0.740

stage.L:age.L -0.17170 0.30201 -0.569 0.570

stage.Q:age.L -0.10578 0.29267 -0.361 0.718

stage.C:age.L -0.42660 0.28324 -1.506 0.132

stage.L:age.Q 0.13072 0.17950 0.728 0.466

stage.Q:age.Q 0.14069 0.17403 0.808 0.419

stage.C:age.Q -0.05505 0.16867 -0.326 0.744

--- Signif. codes: 0 ‘***’ 0.001 ‘**’ 0.01 ‘*’ 0.05 ‘.’ 0.1 ‘ ’ 1

## Mortality after release

Family: binomial ( logit )

Formula: cbind(deaths, N - deaths) ~ release_phase + (1 | year) + (1 | week)

Data: mort_summary %>% filter(release_phase %in% c("SIT 1x", "SIT 2x"))

AIC BIC logLik -2*log(L) df.resid

2469.2 2477.2 -1230.6 2461.2 51

Random effects:

Conditional model:

Groups Name Variance Std.Dev.

year (Intercept) 0.7204 0.8488

week (Intercept) 0.1668 0.4084

Number of obs: 55, groups: year, 2; week, 50

Conditional model:

Estimate Std. Error z value Pr(>|z|)

(Intercept) -2.9943 0.6030 -4.965 6.85e-07 ***

release_phaseSIT 2x -0.0698 0.1171 -0.596 0.551

---Signif. codes: 0 ‘***’ 0.001 ‘**’ 0.01 ‘*’ 0.05 ‘.’ 0.1 ‘ ’ 1

## ETD trends + cyclic seasonal component

Family: Gamma

Link function: log

Formula:

ETD ~ location * phase + s(as.numeric(date), by = location, k = 30) +

s(week_cyc, bs = "cc", k = 20)

Parametric coefficients:

Estimate Std. Error t value Pr(>|t|)

(Intercept) 2.0569 0.3011 6.831 4.83e-11 ***

locationbrasilia -1.2571 0.6464 -1.945 0.0528 .

phasePPF 0.1117 0.3156 0.354 0.7236

phaseBTI 1.0779 0.5134 2.099 0.0366 *

phaseSIT 1x 0.3953 0.6374 0.620 0.5356

phaseSIT 2x 0.3787 0.6869 0.551 0.5818

locationbrasilia:phasePPF 0.3428 0.4785 0.716 0.4743

locationbrasilia:phaseBTI 3.0258 1.2454 2.430 0.0157 *

locationbrasilia:phaseSIT 1x 2.3351 1.4691 1.589 0.1130

locationbrasilia:phaseSIT 2x 2.2818 1.5322 1.489 0.1375

--- Signif. codes: 0 ‘***’ 0.001 ‘**’ 0.01 ‘*’ 0.05 ‘.’ 0.1 ‘ ’ 1

Approximate significance of smooth terms:

edf Ref.df F p-value

s(as.numeric(date)):locationpina 8.431 10.39 3.814 6.86e-05 ***

s(as.numeric(date)):locationbrasilia 27.310 28.64 2.555 4.57e-05 ***

s(week_cyc) 11.761 18.00 2.405 1.36e-06 ***

---

Signif. codes: 0 ‘***’ 0.001 ‘**’ 0.01 ‘*’ 0.05 ‘.’ 0.1 ‘ ’ 1

R-sq.(adj) = 0.58 Deviance explained = 66.9%

GCV = 2.6747 Scale est. = 2.2136 n = 352

## Hatch rate models

Family: beta ( logit )

Formula: hatch_rate ~ location * phase + (1 | year)

Data: hatch_dat

Weights: w

AIC BIC logLik -2*log(L) df.resid

-8663.9 -8630.1 4342.0 -8683.9 208

Random effects:

Conditional model:

Groups Name Variance Std.Dev.

year (Intercept) 0.01081 0.104

Number of obs: 218, groups: year, 3

Dispersion parameter for beta family (): 22.6

Conditional model:

Estimate Std. Error z value Pr(>|z|)

(Intercept) 1.78299 0.07332 24.32 < 2e-16 ***

locationbrasilia -0.36510 0.03868 -9.44 < 2e-16 ***

phaseBTI -2.45968 0.05601 -43.91 < 2e-16 ***

phaseSIT 1x -0.37588 0.06101 -6.16 7.23e-10 ***

phaseSIT 2x -0.14357 0.06759 -2.12 0.0337 *

locationbrasilia:phaseBTI 0.61137 0.04779 12.79 < 2e-16 ***

locationbrasilia:phaseSIT 1x -0.30038 0.04648 -6.46 1.03e-10 ***

locationbrasilia:phaseSIT 2x -1.05819 0.05279 -20.04 < 2e-16 ***

---

Signif. codes: 0 ‘***’ 0.001 ‘**’ 0.01 ‘*’ 0.05 ‘.’ 0.1 ‘ ’ 1

## Recapture model

Family: binomial ( logit )

Formula: recap_rate ~ release_phase + (1 | year) + (1 | week)

Data: recap_mort %>% filter(release_phase %in% c("SIT 1x", "SIT 2x"))

AIC BIC logLik -2*log(L) df.resid

17.8 27.4 -4.9 9.8 77

Random effects:

Conditional model:

Groups Name Variance Std.Dev.

year (Intercept) 9.241e-10 3.040e-05

week (Intercept) 4.882e-15 6.987e-08

Number of obs: 81, groups: year, 2; week, 51

Conditional model:

Estimate Std. Error z value Pr(>|z|)

(Intercept) -4.9450 1.9624 -2.520 0.0117 *

release_phaseSIT 2x 0.7319 2.3307 0.314 0.7535

---

Signif. codes: 0 ‘***’ 0.001 ‘**’ 0.01 ‘*’ 0.05 ‘.’ 0.1 ‘ ’ 1

## Induced sterility model

Family: beta ( logit )

Formula: ind_st ~ location * phase + (1 | year)

Data: ster_dat

Weights: w

AIC BIC logLik -2*log(L) df.resid

-3698.7 -3682.8 1855.3 -3710.7 98

Random effects:

Conditional model:

Groups Name Variance Std.Dev.

year (Intercept) 0.02106 0.1451

Number of obs: 104, groups: year, 2

Dispersion parameter for beta family (): 17.1

Conditional model:

Estimate Std. Error z value Pr(>|z|)

(Intercept) -1.51203 0.10718 -14.107 <2e-16 ***

locationbrasilia 0.05248 0.03400 1.543 0.123

phaseSIT 2x 0.76485 0.04075 18.771 <2e-16 ***

locationbrasilia:phaseSIT 2x -0.04974 0.04953 -1.004 0.315

---

Signif. codes: 0 ‘***’ 0.001 ‘**’ 0.01 ‘*’ 0.05 ‘.’ 0.1 ‘ ’ 1

# Causal Impact

The Causal Impact package offers a report output, which is presented below for each release type and parameter observed.

## 1× release/week

### Wild-type female

During the post-intervention period, the response variable had an average value of approx. 9.37. By contrast, in the absence of an intervention, we would have expected an average response of 11.26. The 95% interval of this counterfactual prediction is [9.66, 12.96]. Subtracting this prediction from the observed response yields an estimate of the causal effect the intervention had on the response variable. This effect is -1.89 with a 95% interval of [-3.59, -0.29]. For a discussion of the significance of this effect, see below.

Summing up the individual data points during the post-intervention period (which can only sometimes be meaningfully interpreted), the response variable had an overall value of 327.99. By contrast, had the intervention not taken place, we would have expected a sum of 394.15. The 95% interval of this prediction is [338.20, 453.64].

The above results are given in terms of absolute numbers. In relative terms, the response variable showed a decrease of -16%. The 95% interval of this percentage is [-28%, -3%].

This means that the negative effect observed during the intervention period is statistically significant. If the experimenter had expected a positive effect, it is recommended to double-check whether anomalies in the control variables may have caused an overly optimistic expectation of what should have happened in the response variable in the absence of the intervention.

The probability of obtaining this effect by chance is very small (Bayesian one-sided tail-area probability p = 0.01). This means the causal effect can be considered statistically significant.

### Egg hatch

During the post-intervention period, the response variable had an average value of approx. 0.67. By contrast, in the absence of an intervention, we would have expected an average response of 0.74. The 95% interval of this counterfactual prediction is [0.69, 0.79]. Subtracting this prediction from the observed response yields an estimate of the causal effect the intervention had on the response variable. This effect is -0.076 with a 95% interval of [-0.13, -0.025]. For a discussion of the significance of this effect, see below.

Summing up the individual data points during the post-intervention period (which can only sometimes be meaningfully interpreted), the response variable had an overall value of 22.05. By contrast, had the intervention not taken place, we would have expected a sum of 24.56. The 95% interval of this prediction is [22.87, 26.23].

The above results are given in terms of absolute numbers. In relative terms, the response variable showed a decrease of -10%. The 95% interval of this percentage is [-16%, -4%].

This means that the negative effect observed during the intervention period is statistically significant. If the experimenter had expected a positive effect, it is recommended to double-check whether anomalies in the control variables may have caused an overly optimistic expectation of what should have happened in the response variable in the absence of the intervention.

The probability of obtaining this effect by chance is very small (Bayesian one-sided tail-area probability p = 0.005). This means the causal effect can be considered statistically significant.

### ETD

During the post-intervention period, the response variable had an average value of approx. 12.70. In the absence of an intervention, we would have expected an average response of 12.52. The 95% interval of this counterfactual prediction is [10.21, 14.80]. Subtracting this prediction from the observed response yields an estimate of the causal effect the intervention had on the response variable. This effect is 0.18 with a 95% interval of [-2.10, 2.49]. For a discussion of the significance of this effect, see below.

Summing up the individual data points during the post-intervention period (which can only sometimes be meaningfully interpreted), the response variable had an overall value of 419.09. Had the intervention not taken place, we would have expected a sum of 413.07. The 95% interval of this prediction is [337.05, 488.42].

The above results are given in terms of absolute numbers. In relative terms, the response variable showed an increase of +3%. The 95% interval of this percentage is [-14%, +24%].

This means that, although the intervention appears to have caused a positive effect, this effect is not statistically significant when considering the entire post-intervention period as a whole. Individual days or shorter stretches within the intervention period may of course still have had a significant effect, as indicated whenever the lower limit of the impact time series (lower plot) was above zero. The apparent effect could be the result of random fluctuations that are unrelated to the intervention. This is often the case when the intervention period is very long and includes much of the time when the effect has already worn off. It can also be the case when the intervention period is too short to distinguish the signal from the noise. Finally, failing to find a significant effect can happen when there are not enough control variables or when these variables do not correlate well with the response variable during the learning period.

The probability of obtaining this effect by chance is p = 0.424. This means the effect may be spurious and would generally not be considered statistically significant.

## 2× releases/week

### Wild-type female

During the post-intervention period, the response variable had an average value of approx. 5.23. By contrast, in the absence of an intervention, we would have expected an average response of 10.79. The 95% interval of this counterfactual prediction is [8.69, 12.85]. Subtracting this prediction from the observed response yields an estimate of the causal effect the intervention had on the response variable. This effect is -5.56 with a 95% interval of [-7.62, -3.46]. For a discussion of the significance of this effect, see below.

Summing up the individual data points during the post-intervention period (which can only sometimes be meaningfully interpreted), the response variable had an overall value of 115.05. By contrast, had the intervention not taken place, we would have expected a sum of 237.33. The 95% interval of this prediction is [191.13, 282.73].

The above results are given in terms of absolute numbers. In relative terms, the response variable showed a decrease of -51%. The 95% interval of this percentage is [-59%, -40%].

This means that the negative effect observed during the intervention period is statistically significant. If the experimenter had expected a positive effect, it is recommended to double-check whether anomalies in the control variables may have caused an overly optimistic expectation of what should have happened in the response variable in the absence of the intervention.

The probability of obtaining this effect by chance is very small (Bayesian one-sided tail-area probability p = 0.001). This means the causal effect can be considered statistically significant.

### Egg hatch

During the post-intervention period, the response variable had an average value of approx. 0.53. By contrast, in the absence of an intervention, we would have expected an average response of 0.78. The 95% interval of this counterfactual prediction is [0.72, 0.85]. Subtracting this prediction from the observed response yields an estimate of the causal effect the intervention had on the response variable. This effect is -0.25 with a 95% interval of [-0.32, -0.19]. For a discussion of the significance of this effect, see below.

Summing up the individual data points during the post-intervention period (which can only sometimes be meaningfully interpreted), the response variable had an overall value of 11.73. By contrast, had the intervention not taken place, we would have expected a sum of 17.26. The 95% interval of this prediction is [15.81, 18.77].

The above results are given in terms of absolute numbers. In relative terms, the response variable showed a decrease of -32%. The 95% interval of this percentage is [-38%, -26%].

This means that the negative effect observed during the intervention period is statistically significant. If the experimenter had expected a positive effect, it is recommended to double-check whether anomalies in the control variables may have caused an overly optimistic expectation of what should have happened in the response variable in the absence of the intervention.

The probability of obtaining this effect by chance is very small (Bayesian one-sided tail-area probability p = 0.001). This means the causal effect can be considered statistically significant.

### ETD

During the post-intervention period, the response variable had an average value of approx. 7.12. By contrast, in the absence of an intervention, we would have expected an average response of 11.99. The 95% interval of this counterfactual prediction is [9.15, 15.14]. Subtracting this prediction from the observed response yields an estimate of the causal effect the intervention had on the response variable. This effect is -4.86 with a 95% interval of [-8.01, -2.03]. For a discussion of the significance of this effect, see below.

Summing up the individual data points during the post-intervention period (which can only sometimes be meaningfully interpreted), the response variable had an overall value of 156.72. By contrast, had the intervention not taken place, we would have expected a sum of 263.73. The 95% interval of this prediction is [201.35, 333.00].

The above results are given in terms of absolute numbers. In relative terms, the response variable showed a decrease of -40%. The 95% interval of this percentage is [-53%, -22%].

This means that the negative effect observed during the intervention period is statistically significant. If the experimenter had expected a positive effect, it is recommended to double-check whether anomalies in the control variables may have caused an overly optimistic expectation of what should have happened in the response variable in the absence of the intervention.

The probability of obtaining this effect by chance is very small (Bayesian one-sided tail-area probability p = 0.004). This means the causal effect can be considered statistically significant.

# R code script

# Recife SIT analysis — optimized, reproducible, and tidy

# -------------------------------------------------------

suppressPackageStartupMessages({

pkgs <- c(

"tidyverse","lubridate","ISOweek","janitor",

"glmmTMB","emmeans","ggeffects","DHARMa","mgcv",

"patchwork","ggpubr","boot","splines","zoo","CausalImpact",

"scales"

)

to_install <- pkgs[!pkgs %in% installed.packages()[,1]]

if (length(to_install)) install.packages(to_install, quiet = TRUE)

lapply(pkgs, require, character.only = TRUE)

})

set.seed(123)

# =========================================================

# 0) Constants & helpers

# =========================================================

PHASE_LEVELS <- c("baseline","PPF","BTI","SIT 1x","SIT 2x","ULV")

PHASE_RANGES <- tibble::tribble(

~phase, ~start, ~end,

"baseline", as.Date("2017-01-23"), as.Date("2019-07-08"),

"PPF", as.Date("2019-07-09"), as.Date("2020-02-09"),

"BTI", as.Date("2020-03-25"), as.Date("2020-09-09"),

"SIT 1x", as.Date("2020-10-27"), as.Date("2021-07-11"),

"SIT 2x", as.Date("2021-07-12"), as.Date("2021-12-12")

) %>% mutate(phase = factor(phase, levels = PHASE_LEVELS))

KEY_EVENTS <- tibble::tribble(

~date, ~label,

as.Date("2019-07-09"), "PPF",

as.Date("2020-03-25"), "BTI",

as.Date("2020-10-27"), "Release 1×",

as.Date("2021-07-12"), "Release 2×"

)

map_phase <- function(d) {

case_when(

d >= ymd("2017-01-23") & d <= ymd("2019-07-08") ~ "baseline",

d >= ymd("2019-07-09") & d <= ymd("2020-02-09") ~ "PPF",

d >= ymd("2020-03-25") & d <= ymd("2020-09-09") ~ "BTI",

d >= ymd("2020-10-27") & d <= ymd("2021-07-11") ~ "SIT 1x",

d >= ymd("2021-07-12") & d <= ymd("2021-12-12") ~ "SIT 2x",

d %in% ymd(c("2020-08-24","2020-08-31","2020-06-21","2021-06-28","2021-07-05")) ~ "ULV",

TRUE ~ NA_character_

)

}

fmt_ci <- function(est, lcl, ucl, digits = 2, percent = FALSE) {

if (percent) {

sprintf("%s [%s–%s]",

scales::percent(est, accuracy = 0.1),

scales::percent(lcl, accuracy = 0.1),

scales::percent(ucl, accuracy = 0.1))

} else {

sprintf(paste0("%.", digits, "f [%.", digits, "f–%.", digits, "f]"), est, lcl, ucl)

}

}

# Plot aesthetics

PAL_LOC <- c('pina'="#00FFA5", 'brasilia'="#A500FF")

RIB_COL <- "gray95"

MAIN_1 <- "#00FFA5"; MAIN_2 <- "#A500FF"

PRED_LTY <- "longdash"; INT_LTY <- "dotted"

theme_custom <- theme_classic() +

theme(plot.title.position = "panel",

legend.position = "bottom",

axis.line = element_line(color = "gray20"),

panel.grid.major.x = element_blank())

# =========================================================

# 1) Data import & harmonization

# =========================================================

releases <- read_csv("./releases.csv", show_col_types = FALSE) %>%

drop_na(total) %>%

mutate(

release = dmy(release),

year = epiyear(release),

week = epiweek(release),

month = month(release),

release_phase = map_phase(release),

area = case_when(

release_phase == "baseline" ~ 58,

release_phase %in% c("SIT 1x","SIT 2x") ~ 60,

TRUE ~ 118

),

interact = interaction(year, week, release_phase)

) %>%

filter(release <= ymd("2021-12-12")) %>%

group_by(release, year, week, release_phase, interact, area) %>%

reframe(m_release = sum(total), .groups = "drop") %>%

mutate(release_ha = m_release / area)

ovitrap <- read_csv("ovitrap.csv", show_col_types = FALSE) %>%

mutate(

iso_str = sprintf("%d-W%02d-1", year, week),

date = ISOweek2date(iso_str),

location = factor(location, levels = c("pina","brasilia")),

phase = factor(map_phase(date), levels = PHASE_LEVELS),

w = ifelse(is.na(trap_counting) | trap_counting <= 0, 1, trap_counting)

) %>%

filter(date <= ymd("2021-12-12"))

bgstrap <- read_csv("./bgtrap.csv", show_col_types = FALSE) %>%

mutate(

date = dmy(date),

year = epiyear(date),

week = epiweek(date),

month = month(date),

location = factor(location, levels = c("pina","brasilia")),

release_phase = map_phase(date)

) %>%

# pivot male/female x wild/sterile safely (avoid hard-coded column indices)

pivot_longer(cols = matches("^(male|female)_(wild|sterile)$"),

names_to = "a_type", values_to = "total_adult") %>%

separate(a_type, into = c("sex","type"), sep = "_") %>%

filter(date <= ymd("2021-12-12"))

mortality <- read_csv("./release_mortality.csv", show_col_types = FALSE) %>%

mutate(

date = ymd(date),

year = epiyear(date),

week = epiweek(date),

release_phase = map_phase(date),

interact = interaction(year, week, release_phase)

) %>%

filter(date <= ymd("2021-12-12"))

# =========================================================

# 2) ETD (GAM trend) + base ETD plot

# =========================================================

ovitrap2 <- ovitrap %>%

mutate(week_cyc = pmin(isoweek(date), 52)) %>%

filter(!is.na(phase), !is.na(ETD), !is.na(date), !is.na(week))

# Trend + cyclic seasonal component

set.seed(123)

gam_etd <- gam(

ETD ~ location * phase +

s(as.numeric(date), by = location, k = 30) +

s(week_cyc, bs = "cc", k = 20),

data = ovitrap2,

family = Gamma(link = "log"),

weights = w,

knots = list(week_cyc = c(0.5, 52.5))

)

newd <- ovitrap2 %>%

distinct(location, phase) %>%

tidyr::crossing(date = seq(min(ovitrap2$date), max(ovitrap2$date), by = "1 week")) %>%

left_join(PHASE_RANGES, by = "phase") %>%

filter(!is.na(start), date >= start, date <= end) %>%

mutate(week_cyc = pmin(isoweek(date), 52))

pr <- predict(gam_etd, newdata = newd, type = "link", se.fit = TRUE)

plot_df <- newd %>%

mutate(fit = exp(pr$fit),

lwr = exp(pr$fit - 1.96*pr$se.fit),

upr = exp(pr$fit + 1.96*pr$se.fit))

ETD_plot <- ggplot() +

geom_line(data = ovitrap2, aes(date, ETD, color = location), alpha = 0.35) +

geom_ribbon(data = plot_df, aes(date, ymin = lwr, ymax = upr, fill = location), alpha = 0.18) +

geom_line(data = plot_df, aes(date, fit, color = location), linewidth = 1) +

geom_vline(data = KEY_EVENTS, aes(xintercept = as.numeric(date)), linetype = 2, alpha = 0.6) +

geom_text(data = KEY_EVENTS, aes(x = date - 60, y = 53, label = label), vjust = 1.2, size = 3, angle = 90) +

scale_y_continuous("Eggs / Trap / Day (ETD)", labels = label_number(accuracy = 1)) +

scale_color_manual(name = "", values = PAL_LOC, labels = c('pina' = "Pina", 'brasilia' = "B.Teimosa")) +

scale_fill_manual(name = "", values = PAL_LOC, labels = c('pina' = "Pina", 'brasilia' = "B.Teimosa")) +

scale_x_date(NULL) +

theme_classic(base_size = 12) +

theme(legend.position = c(0.2, 0.8))

# A simpler ETD time-series with release bars

ovitrap_plot <- ggplot() +

geom_bar(data = releases, aes(x = release, y = m_release/10000, fill = release_phase),

stat = "identity", width = 2.5, alpha = 0.2) +

geom_vline(data = KEY_EVENTS, aes(xintercept = as.numeric(date)), linetype = 2, alpha = 0.6) +

geom_text(data = KEY_EVENTS, aes(x = date - 60, y = 40, label = label), vjust = 1.2, angle = 90) +

stat_summary(data = ovitrap, aes(x = date, y = ETD, col = location), fun = mean, geom = "line", linewidth = 1) +

labs(x = "Month / Year", y = "Eggs/Trap/Day - ETD") +

scale_x_date(date_breaks = "6 month", date_labels = "%m/%y", expand = c(0.02, 0.02)) +

scale_y_continuous(breaks = seq(0, 60, by = 10),

sec.axis = sec_axis(~.*10, breaks = seq(0, 600, by = 50), name = "Sterile male released (× thousand)"),

expand = c(0.01, 0.01)) +

scale_fill_manual(name = "", values = c(PAL_LOC, "SIT 1x" = "gray70", "SIT 2x" = "gray40"),

labels = c('pina' = "Pina", 'brasilia' = "Brasilia Teimosa", "SIT 1x" = "Release 1×", "SIT 2x" = "Release 2×")) +

scale_color_manual(name = "", values = c(PAL_LOC, "SIT 1x" = "gray70", "SIT 2x" = "gray40"),

labels = c('pina' = "Pina", 'brasilia' = "Brasilia Teimosa", "SIT 1x" = "Release 1×", "SIT 2x" = "Release 2×")) +

theme_classic(base_size = 14) +

theme(legend.position = c(0.2,0.85), legend.direction = "horizontal",

axis.text = element_text(size = 14, colour = "black"), legend.text = element_text(size = 14, colour = "black"))

# =========================================================

# 3) Hatch rate models & plot

# =========================================================

hatch_dat <- ovitrap %>%

filter(!is.na(hatch_rate), phase %in% c("baseline","PPF","BTI","SIT 1x","SIT 2x")) %>%

mutate(

phase = factor(phase, levels = c("baseline","PPF","BTI","SIT 1x","SIT 2x")),

location = factor(location, levels = c("pina","brasilia")),

hatch_rate = pmin(pmax(hatch_rate, 1e-4), 1-1e-4),

larva = n_eggs*hatch_rate

)

mod_hatch <- glmmTMB(

hatch_rate ~ location * phase + (1 | year),

data = hatch_dat,

family = beta_family(link = "logit"),

weights = w

)

emm_hatch <- emmeans(mod_hatch, ~ location | phase, type = "response")

emm_hatch_resp <- regrid(emmeans(mod_hatch, ~ location | phase, data = hatch_dat))

hatch_plot <- ggplot() +

geom_bar(data = releases, aes(x = release, y = m_release/1000000, fill = release_phase),

stat = "identity", width = 2.5, alpha = 0.2) +

stat_summary(data = ovitrap %>% filter(phase %in% c("SIT 1x","SIT 2x")),

aes(x = date, y = hatch_rate, col = location), fun = mean, geom = "line", linewidth = 1) +

labs(x = "Month / Year", y = "Egg Hatch") +

geom_vline(data = KEY_EVENTS[c(3,4),], aes(xintercept = as.numeric(date)), linetype = 2, alpha = 0.6) +

geom_text(data = KEY_EVENTS[c(3,4),], aes(x = date + 20, y = 1, label = label), vjust = 1.2) +

scale_x_date(date_breaks = "2 month", date_labels = "%m/%y", expand = c(0.02, 0.02)) +

scale_y_continuous(breaks = seq(0, 1, by = 0.2), labels = percent_format(accuracy = 1),

sec.axis = sec_axis(~.*1, breaks = seq(0, 1, by = 0.2), name = "Sterile male released (× million)"),

expand = c(0.01, 0.01)) +

scale_fill_manual(name = "", values = c(PAL_LOC, "SIT 1x" = "gray70", "SIT 2x" = "gray40"),

labels = c('pina' = "Pina", 'brasilia' = "Brasilia Teimosa", "SIT 1x" = "Release 1×", "SIT 2x" = "Release 2×")) +

scale_color_manual(name = "", values = c(PAL_LOC, "SIT 1x" = "gray70", "SIT 2x" = "gray40"),

labels = c('pina' = "Pina", 'brasilia' = "Brasilia Teimosa", "SIT 1x" = "Release 1×", "SIT 2x" = "Release 2×")) +

theme_classic(base_size = 14) +

theme(legend.position = c(0.3,0.1), legend.direction = "horizontal",

axis.text = element_text(size = 14, colour = "black"), legend.text = element_text(size = 14, colour = "black"))

# =========================================================

# 4) BG trap — recapture, mortality, ratio, plots

# =========================================================

# Recapture (weekly sterile males captured vs released)

bg_week_ster <- bgstrap %>%

filter(sex == "male", type == "sterile") %>%

mutate(interact = interaction(year, week, release_phase)) %>%

group_by(year, week, date, release_phase, interact) %>%

drop_na(total_adult) %>%

reframe(m_captured = sum(total_adult), .groups = "drop")

bg_recap <- bg_week_ster %>%

left_join(releases %>% select(interact, m_release), by = "interact") %>%

mutate(recap_rate = (m_captured * 2) / m_release) %>%

select(year, week, date, release_phase, recap_rate)

# Mortality per batch -> weekly summary

mort_week <- mortality %>%

group_by(date, year, week, release_phase, release_batch) %>%

summarise(

deaths_batch = sum(total_death, na.rm = TRUE),

reps = n(),

n_total = 1000 * reps, # adjust if n_per_replicate != 1000

mort_prop = deaths_batch / n_total,

.groups = "drop"

) %>%

mutate(phase = factor(release_phase, levels = c("SIT 1x","SIT 2x","BTI","PPF","baseline","ULV")))

mort_summary <- mort_week %>%

group_by(year, week, release_phase) %>%

summarise(

deaths = sum(deaths_batch, na.rm = TRUE),

N = sum(n_total, na.rm = TRUE),

mort_p = deaths / pmax(N, 1),

date_w = min(date, na.rm = TRUE),

.groups = "drop"

) %>%

mutate(phase = factor(release_phase, levels = c("SIT 1x","SIT 2x","BTI","PPF","baseline","ULV")))

# QC ribbon for mortality

p_qc <- mort_summary %>%

filter(release_phase %in% c("SIT 1x","SIT 2x")) %>%

mutate(se = sqrt((mort_p*(1-mort_p))/pmax(N,1)),

lwr = pmax(0, mort_p - 1.96*se),

upr = pmin(1, mort_p + 1.96*se)) %>%

ggplot(aes(date_w, mort_p, color = release_phase)) +

geom_line() + geom_point() +

geom_ribbon(aes(ymin = lwr, ymax = upr, fill = release_phase), alpha = 0.15, color = NA) +

scale_y_continuous("Mortality of shipped males", labels = percent) +

scale_x_date(NULL) + theme_bw(base_size = 12) + theme(legend.position = "top")

# Recap vs Mort scatter

recap_mort <- bg_recap %>%

left_join(mort_summary %>% select(year, week, mort_p, release_phase), by = c("year","week","release_phase"))

# Model recap ~ phase (random year/week)

mod_recap <- glmmTMB(

recap_rate ~ release_phase + (1|year) + (1|week),

data = recap_mort %>% filter(release_phase %in% c("SIT 1x","SIT 2x")),

family = binomial(link = "logit")

)

# Wild females time series with release bars

bgstrap_plot <- ggplot() +

geom_bar(data = releases, aes(x = release, y = m_release/10000, fill = release_phase),

stat = "identity", width = 2.5, alpha = 0.2) +

stat_summary(data = bgstrap %>% filter(sex=="female", type=="wild", date >= ymd("2019-04-01")),

aes(x = date, y = total_adult, col = location), fun = mean, geom = "line", linewidth = 1) +

geom_vline(data = KEY_EVENTS, aes(xintercept = as.numeric(date)), linetype = 2, alpha = 0.6) +

geom_text(data = KEY_EVENTS, aes(x = date - 20, y = 40, label = label), vjust = 1.2, angle = 90) +

labs(x = "Month / Year", y = "Number of wild females") +

scale_x_date(date_breaks = "4 month", date_labels = "%m/%y", expand = c(0.02, 0.02)) +

scale_y_continuous(breaks = seq(0, 60, by = 10),

sec.axis = sec_axis(~.*10, breaks = seq(0, 600, by = 50), name = "Sterile male released (× thousand)"),

expand = c(0.01, 0.01)) +

scale_fill_manual(name = "", values = c(PAL_LOC, "SIT 1x" = "gray70", "SIT 2x" = "gray40"),

labels = c('pina' = "Pina", 'brasilia' = "Brasilia Teimosa", "SIT 1x" = "Release 1×", "SIT 2x" = "Release 2×")) +

scale_color_manual(name = "", values = c(PAL_LOC, "SIT 1x" = "gray70", "SIT 2x" = "gray40"),

labels = c('pina' = "Pina", 'brasilia' = "Brasilia Teimosa", "SIT 1x" = "Release 1×", "SIT 2x" = "Release 2×")) +

theme_classic(base_size = 14) +

theme(legend.position = c(0.3,0.75), legend.direction = "horizontal",

axis.text = element_text(size = 14, colour = "black"), legend.text = element_text(size = 14, colour = "black"))

mort_release_plot <- ggplot() +

geom_bar(data = releases, aes(x = release, y = m_release/1000000, fill = release_phase),

stat = "identity", width = 3, alpha = 0.2) +

stat_summary(data = recap_mort, aes(x = date, y = recap_rate), linetype = "dashed",

fun = mean, geom = "line", linewidth = 1, color = "#A500FF") +

stat_summary(data = mort_summary, aes(x = date_w, y = mort_p),

fun = mean, geom = "line", linewidth = 1, color = "#00FFA5") +

scale_x_date(name = "Month / Year", date_breaks = "10 week", date_labels = "%m/%y", expand = c(0.02, 0.02)) +

scale_y_continuous(name = "Mortality & Recapture", breaks = seq(0, 1, by = 0.05), labels = percent_format(accuracy = 1),

sec.axis = sec_axis(~.*1000, breaks = seq(0, 600, by = 50), name = "Sterile Male Released (× thousand)"),

expand = c(0.01, 0.01)) +

scale_fill_manual(name = "", values = c("SIT 1x" = "gray70", "SIT 2x" = "gray40"), labels = c("SIT 1x" = "Release 1×", "SIT 2x" = "Release 2×")) +

theme_classic(base_size = 12) +

theme(legend.position = c(0.3,0.8), legend.direction = "horizontal",

axis.text = element_text(size = 12, colour = "black"), legend.text = element_text(size = 12, colour = "black"))

# =========================================================

# 5) Induced sterility & ST:WT ratio (models + plots)

# =========================================================

ster_dat_raw <- ovitrap %>%

filter(phase %in% c("SIT 1x","SIT 2x"), !is.na(induced_sterility)) %>%

mutate(

phase = factor(phase, levels = c("SIT 1x","SIT 2x")),

location = factor(location, levels = c("pina","brasilia"))

)

ster_dat <- ster_dat_raw %>%

mutate(ind_st = induced_sterility,

ind_st = ifelse(ind_st > 1 & ind_st <= 100, ind_st/100, ind_st),

ind_st = ifelse(ind_st < 0 | ind_st > 1, NA_real_, ind_st),

ind_st = pmin(pmax(ind_st, 1e-4), 1 - 1e-4),

w = ifelse(is.na(trap_counting) | trap_counting <= 0, 1, trap_counting)) %>%

filter(!is.na(ind_st))

mod_sterility <- glmmTMB(

ind_st ~ location * phase + (1 | year),

data = ster_dat,

family = beta_family(link = "logit"),

weights = w

)

emm_ster <- emmeans(mod_sterility, ~ location | phase, type = "response")

emm_ster_resp <- regrid(emmeans(mod_sterility, ~ location | phase))

induced_sterility_plot <- ggplot() +

geom_bar(data = releases, aes(x = release, y = m_release/1000000, fill = release_phase),

stat = "identity", width = 3, alpha = 0.2) +

stat_summary(data = ster_dat_raw %>% filter(induced_sterility > 0, location == "brasilia"),

aes(x = date, y = induced_sterility/100), fun = mean, geom = "line",

linetype = 2, linewidth = 1, col = "#A500FF") +

geom_smooth(data = ster_dat_raw %>% filter(induced_sterility > 0, location == "brasilia"),

aes(x = date, y = induced_sterility/100), col = "#00FFA5",

method = "glm", method.args = list(family = "binomial"), se = TRUE, alpha = .1) +

labs(x = "Month / Year", y = "Induced Sterility (%)") +

scale_x_date(date_breaks = "3 month", date_labels = "%m/%y", expand = c(0.02, 0.02)) +

scale_y_continuous(breaks = seq(0, 1, by = .2), labels = percent_format(accuracy = 1),

sec.axis = sec_axis(~.*1000, breaks = seq(0, 600, by = 100), name = "Sterile male released (× thousand)"),

expand = c(0.01, 0.01)) +

scale_fill_manual(name = "", values = c(PAL_LOC, "SIT 1x" = "gray70", "SIT 2x" = "gray40"),

labels = c('pina' = "Pina", 'brasilia' = "Brasilia Teimosa", "SIT 1x" = "Release 1×", "SIT 2x" = "Release 2×")) +

scale_color_manual(name = "", values = c(PAL_LOC, "SIT 1x" = "gray70", "SIT 2x" = "gray40"),

labels = c('pina' = "Pina", 'brasilia' = "Brasilia Teimosa", "SIT 1x" = "Release 1×", "SIT 2x" = "Release 2×")) +

theme_classic(base_size = 12) +

theme(legend.position = c(0.3,0.85), legend.direction = "horizontal",

axis.text = element_text(size = 12, colour = "black"), legend.text = element_text(size = 12, colour = "black"))

# Sterile:Wild ratio — compute weekly ratio dataset

ratio_week <- bgstrap %>%

filter(sex == "male", type %in% c("wild","sterile"), !is.na(total_adult)) %>%

group_by(location, year, week, date, release_phase, type) %>%

summarise(n = sum(total_adult), .groups = "drop_last") %>%

pivot_wider(names_from = type, values_from = n, values_fill = 0) %>%

ungroup() %>%

mutate(

wild = pmax(wild, 0),

sterile = pmax(sterile, 0),

sterile_wild = ifelse(wild == 0 & sterile == 0, 0, sterile / pmax(wild, 1e-6)),

log_ratio = log1p(sterile_wild)

)

# Gaussian on log(1 + ratio)

mod_ratio_ln <- glmmTMB(

log_ratio ~ location * release_phase + (1|year) + (1|week),

data = ratio_week %>% filter(release_phase %in% c("SIT 1x","SIT 2x")),

family = gaussian(link = "identity")

)

st_wt_plot <- ggplot() +

geom_bar(data = releases, aes(x = release, y = m_release/10000, fill = release_phase),

stat = "identity", width = 3, alpha = 0.2) +

stat_summary(data = ratio_week %>% filter(release_phase %in% c("SIT 1x","SIT 2x"), location == "brasilia"),

aes(x = date, y = sterile_wild), fun = mean, geom = "line", linewidth = 1, col = "#A500FF") +

labs(x = "Month / Year", y = "Sterile to Wild ratio") +

scale_x_date(date_breaks = "3 month", date_labels = "%m/%y", expand = c(0.02, 0.02)) +

scale_y_continuous(breaks = seq(0, 60, by = 10), sec.axis = sec_axis(~.*10, breaks = seq(0, 600, by = 100), name = "Sterile male released (× thousand)"), expand = c(0.01, 0.01)) +

scale_fill_manual(name = "", values = c(PAL_LOC, "SIT 1x" = "gray70", "SIT 2x" = "gray40"),

labels = c('pina' = "Pina", 'brasilia' = "Brasilia Teimosa", "SIT 1x" = "Release 1×", "SIT 2x" = "Release 2×")) +

scale_color_manual(name = "", values = c(PAL_LOC, "SIT 1x" = "gray70", "SIT 2x" = "gray40"),

labels = c('pina' = "Pina", 'brasilia' = "Brasilia Teimosa", "SIT 1x" = "Release 1×", "SIT 2x" = "Release 2×")) +

theme_classic(base_size = 12) +

theme(legend.position = c(0.3,0.85), legend.direction = "horizontal",

axis.text = element_text(size = 12, colour = "black"), legend.text = element_text(size = 12, colour = "black"))

# =========================================================

# 6) Flight ability (betabinomial) + plots

# =========================================================

flight_ability <- read_csv("./flight_ability.csv", show_col_types = FALSE) %>%

mutate(

date = dmy(date),

year = epiyear(date),

week = epiweek(date),

age = factor(age, ordered = TRUE, levels = c("72-96h","96 -120 h","120 -144 h")),

escape_rate = final_total / initial_total,

escaped = final_total,

not_escaped = initial_total - final_total,

group = ordered(group, levels = c("control","chilling","chilling_compaction_transport","chilling_compaction_transport_irradiation_marking")),

stage = factor(case_when(

group == "control" ~ "I",

group == "chilling" ~ "II",

group == "chilling_compaction_transport" ~ "III",

group == "chilling_compaction_transport_irradiation_marking" ~ "IV"),

ordered = TRUE, levels = c("I","II","III","IV"))

)

flight_f <- flight_ability %>%

filter(type == "fliers") %>%

mutate(

escaped = final_total,

not_escaped = initial_total - final_total,

escape_rate = escaped / initial_total,

stage = factor(case_when(

group == "control" ~ "I",

group == "chilling" ~ "II",

group == "chilling_compaction_transport" ~ "III",

group == "chilling_compaction_transport_irradiation_marking" ~ "IV",

TRUE ~ NA_character_

), levels = c("I","II","III","IV"), ordered = TRUE),

age = factor(age, levels = c("72-96h","96 -120 h","120 -144 h"), ordered = TRUE)

)

stopifnot(all(flight_f$escaped + flight_f$not_escaped == flight_f$initial_total))

m_cat <- glmmTMB(

cbind(escaped, not_escaped) ~ stage * age + (1 | release_batch),

family = betabinomial(link = "logit"),

data = flight_f

)

emm_cat <- emmeans(m_cat, ~ stage | age, type = "response")

pred_phi <- ggpredict(m_cat, terms = c("stage","age")) %>%

as.data.frame() %>%

rename(stage = x, age = group) %>%

mutate(

stage = factor(stage, levels = levels(flight_f$stage), ordered = TRUE),

age = factor(age, levels = levels(flight_f$age), ordered = TRUE),

label = percent(predicted, accuracy = 0.1)

)

pal_stage <- c(I = "gray80", II = "gray60", III = "gray40", IV = "gray20")

p_box_age_pred <- ggplot(flight_f, aes(x = stage, y = escape_rate)) +

geom_boxplot(aes(fill = stage), width = 0.65, outlier.shape = NA, alpha = 0.85) +

geom_jitter(aes(color = stage), width = 0.10, alpha = 0.15, size = 0.9) +

geom_text(data = pred_phi, aes(x = stage, y = 1, label = label), angle = 45, inherit.aes = FALSE, vjust = 0, size = 3, fontface = 1) +

facet_grid(~ age) +

scale_y_continuous(labels = percent_format(accuracy = 1), limits = c(0,1)) +

scale_fill_manual(values = pal_stage, drop = FALSE) +

scale_color_manual(values = pal_stage, drop = FALSE) +

labs(x = "Handling stage (I–IV)", y = "Escaping rate") +

theme_classic(base_size = 12) +

theme(legend.position = "none", plot.title.position = "plot", strip.background = element_rect(fill = "white", color = "white"))

# Supplementary EMM figure

flight_emm <- as.data.frame(emm_cat) %>%

mutate(stage = factor(stage, levels = c("I","II","III","IV"), ordered = TRUE),

age = factor(age, levels = c("72-96h","96 -120 h","120 -144 h"), ordered = TRUE),

label = percent(prob, accuracy = 0.1))

p_emm <- ggplot(flight_emm, aes(x = stage, y = prob, color = stage)) +

geom_point(size = 2) +

geom_errorbar(aes(ymin = asymp.LCL, ymax = asymp.UCL), width = 0.16) +

geom_line(aes(group = 1), linewidth = 0.6, color = "grey40") +

geom_text(aes(label = label, y = pmin(prob + 0.06, 0.98)), color = "black", size = 3.3, vjust = 0, fontface = 2) +

facet_wrap(~ age, nrow = 1) +

scale_y_continuous(labels = percent_format(accuracy = 1), limits = c(0,1)) +

scale_color_manual(values = pal_stage, guide = "none") +

labs(x = "Stage (I–IV)", y = "Model-predicted escape rate", title = "Predicted flight ability by stage and age") +

theme_bw(base_size = 12) +

theme(plot.title.position = "plot")

# =========================================================

# 7) Paper figures (04 & 05)

# =========================================================

recife_plot <- ggarrange(ovitrap_plot, hatch_plot, bgstrap_plot, ncol = 1, nrow = 3, labels = "AUTO", common.legend = TRUE, legend = "bottom")

transport_plot <- ggarrange(p_box_age_pred, mort_release_plot, induced_sterility_plot, st_wt_plot, align = "hv", labels = "AUTO", nrow = 2, ncol = 2)

# =========================================================

# 8) Causal Impact (adult, ETD, hatch) + combined figure 06

# =========================================================

pre_period_1 <- c(ymd("2019-05-07"), ymd("2020-10-26"))

post_period_1 <- c(ymd("2020-10-27"), ymd("2021-06-20"))

pre_period_2 <- c(ymd("2019-05-07"), ymd("2020-07-11"))

post_period_2 <- c(ymd("2021-07-12"), ymd("2021-12-12"))

pre_period_11 <- c(ymd("2017-04-01"), ymd("2020-10-26"))

pre_period_21 <- c(ymd("2017-04-01"), ymd("2020-07-11"))

# Adult females

bg_causal <- bgstrap %>%

filter(type == "wild", sex == "female", date <= ymd("2021-12-12")) %>%

group_by(location, date) %>%

summarise(total_adult = mean(total_adult, na.rm = TRUE), .groups = "drop") %>%

pivot_wider(names_from = location, values_from = total_adult) %>%

mutate(across(c(pina, brasilia), ~replace_na(., 0)))

bg_z <- zoo(cbind(SIT = bg_causal$brasilia, Pina = bg_causal$pina), order.by = bg_causal$date)

impact_1 <- CausalImpact(data = bg_z, pre.period = pre_period_1, post.period = post_period_1)

impact_2 <- CausalImpact(data = bg_z, pre.period = pre_period_2, post.period = post_period_2)

impact_1_plot <- plot(impact_1) + theme_classic() + theme(strip.background = element_blank())

impact_2_plot <- plot(impact_2) + theme_classic() + theme(strip.background = element_blank())

# ETD (ovitrap)

ov_causal <- ovitrap %>%

group_by(location, date) %>%

summarise(total_eggs = mean(ETD, na.rm = TRUE), .groups = "drop") %>%

pivot_wider(names_from = location, values_from = total_eggs) %>%

filter(!is.na(date)) %>%

mutate(across(c(pina, brasilia), ~replace_na(., 0)))

ov_z <- zoo(cbind(SIT = ov_causal$brasilia, Pina = ov_causal$pina), order.by = ov_causal$date)

egg_impact_1 <- CausalImpact(data = ov_z, pre.period = pre_period_11, post.period = post_period_1)

egg_impact_2 <- CausalImpact(data = ov_z, pre.period = pre_period_21, post.period = post_period_2)

egg_impact_1_plot <- plot(egg_impact_1) + theme_classic() + theme(strip.background = element_blank())

egg_impact_2_plot <- plot(egg_impact_2) + theme_classic() + theme(strip.background = element_blank())

# Hatch rate

hr_causal <- ovitrap %>%

group_by(location, date) %>%

summarise(total_hatch = mean(hatch_rate, na.rm = TRUE), .groups = "drop") %>%

pivot_wider(names_from = location, values_from = total_hatch) %>%

filter(complete.cases(.)) %>%

mutate(across(c(pina, brasilia), ~replace_na(., 0)))

hr_z <- zoo(cbind(SIT = hr_causal$brasilia, Pina = hr_causal$pina), order.by = hr_causal$date)

hatch_impact_1 <- CausalImpact(data = hr_z, pre.period = pre_period_1, post.period = post_period_1)

hatch_impact_2 <- CausalImpact(data = hr_z, pre.period = pre_period_2, post.period = post_period_2)

hatch_impact_1_plot <- plot(hatch_impact_1) + theme_classic() + theme(strip.background = element_blank())

hatch_impact_2_plot <- plot(hatch_impact_2) + theme_classic() + theme(strip.background = element_blank())

# Styled originals (adult, eggs, hatch) for 1× and 2×

style_ci <- function(ci_obj, main_col) {

df <- as.data.frame(ci_obj$series) %>% tibble::rownames_to_column("date") %>% mutate(date = as.Date(date))

ggplot(df, aes(x = date)) +

geom_ribbon(aes(ymin = point.pred.lower, ymax = point.pred.upper), alpha = 0.9, fill = RIB_COL) +

geom_vline(aes(xintercept = ci_obj$model$post.period[1]), color = "darkred", linetype = INT_LTY, linewidth = 0.8) +

geom_line(aes(y = point.pred, group = 1), color = "gray30", linetype = PRED_LTY, linewidth = 0.6) +

geom_line(aes(y = response, group = 1), color = main_col, linewidth = 0.8) +

scale_x_date(date_breaks = "20 week", date_labels = "%m/%y") + theme_custom

}

gg_original_adult_1 <- style_ci(impact_1, MAIN_1) + labs(x = "Month/Year", y = "Wild Female Mosquito Population")

gg_original_adult_2 <- style_ci(impact_2, MAIN_2) + labs(x = "Month/Year", y = "Wild Female Mosquito Population")

gg_original_eggs_1 <- style_ci(egg_impact_1, MAIN_1) + labs(x = "Month/Year", y = "N. Eggs/Trap/Day - ETD") + scale_x_date(date_breaks = "40 week", date_labels = "%m/%y")

gg_original_eggs_2 <- style_ci(egg_impact_2, MAIN_2) + labs(x = "Month/Year", y = "N. Eggs/Trap/Day - ETD") + scale_x_date(date_breaks = "40 week", date_labels = "%m/%y")

gg_original_hatch_1 <- style_ci(hatch_impact_1, MAIN_1) + labs(x = "Month/Year", y = "Hatch Rate")

gg_original_hatch_2 <- style_ci(hatch_impact_2, MAIN_2) + labs(x = "Month/Year", y = "Hatch Rate")

# Cumulative effects for ribbons

cum_effect_df <- function(ci_obj, phase_label) {

as.data.frame(ci_obj$series) %>%

tibble::rownames_to_column("date") %>% mutate(date = as.Date(date)) %>%

transmute(date, cum.effect, cum.effect.lower, cum.effect.upper, release_phase = phase_label)

}

sit1x_eggs <- cum_effect_df(egg_impact_1, "SIT 1x") %>% filter(date >= ymd("2020-10-01"))

sit2x_eggs <- cum_effect_df(egg_impact_2, "SIT 2x") %>% filter(date >= ymd("2021-07-01"))

sit1x_hatch <- cum_effect_df(hatch_impact_1, "SIT 1x") %>% filter(date >= ymd("2020-10-01")) %>% mutate(parameter = "hatch")

sit2x_hatch <- cum_effect_df(hatch_impact_2, "SIT 2x") %>% filter(date >= ymd("2021-07-01")) %>% mutate(parameter = "hatch")

sit1x_adult <- cum_effect_df(impact_1, "SIT 1x") %>% filter(date >= ymd("2020-10-01")) %>% mutate(parameter = "adult")

sit2x_adult <- cum_effect_df(impact_2, "SIT 2x") %>% filter(date >= ymd("2021-07-01")) %>% mutate(parameter = "adult")

# Eggs cumulative effect comparison

effect_plot_eggs_phase <- bind_rows(sit1x_eggs, sit2x_eggs) %>%

drop_na(cum.effect) %>% mutate(release_phase = factor(release_phase, levels = c("SIT 1x","SIT 2x"))) %>%

ggplot(aes(x = date, fill = release_phase, color = release_phase)) +

geom_ribbon(aes(ymin = cum.effect.lower, ymax = cum.effect.upper), alpha = 0.2) +

geom_vline(xintercept = ymd(c("2019-07-09","2020-03-25","2020-10-27","2021-07-12")), color = "darkred", linetype = INT_LTY, linewidth = 0.8) +

geom_line(aes(y = cum.effect, group = 1)) + geom_hline(yintercept = 0) +

labs(x = "Month/Year", y = "Cumulative Causal Effect") +

scale_y_continuous(expand = c(0,0)) + scale_x_date(date_breaks = "10 week", date_labels = "%m/%y") +

scale_color_manual(name = "", values = c("SIT 1x" = MAIN_1, "SIT 2x" = MAIN_2), labels = c("SIT 1x" = "Release 1×", "SIT 2x" = "Release 2×")) +

scale_fill_manual(name = "", values = c("SIT 1x" = MAIN_1, "SIT 2x" = MAIN_2), labels = c("SIT 1x" = "Release 1×", "SIT 2x" = "Release 2×")) +

theme_classic() + theme(legend.position = c(0.2,0.1))

# Adult cumulative effect

effect_plot_adult_phase <- bind_rows(sit1x_adult, sit2x_adult) %>%

drop_na(cum.effect) %>% mutate(release_phase = factor(release_phase, levels = c("SIT 1x","SIT 2x"))) %>%

ggplot(aes(x = date, fill = release_phase, color = release_phase)) +

geom_ribbon(aes(ymin = cum.effect.lower, ymax = cum.effect.upper), alpha = 0.2) +

geom_vline(xintercept = ymd(c("2020-10-27","2021-07-12")), color = "darkred", linetype = INT_LTY, linewidth = 0.8) +

geom_line(aes(y = cum.effect, group = 1)) + geom_hline(yintercept = 0) +

labs(x = "Month/Year", y = "Cumulative Causal Effect") +

scale_y_continuous(expand = c(0,0)) + scale_x_date(date_breaks = "10 week", date_labels = "%m/%y") +

scale_color_manual(name = "", values = c("SIT 1x" = MAIN_1, "SIT 2x" = MAIN_2), labels = c("SIT 1x" = "Release 1×", "SIT 2x" = "Release 2×")) +

scale_fill_manual(name = "", values = c("SIT 1x" = MAIN_1, "SIT 2x" = MAIN_2), labels = c("SIT 1x" = "Release 1×", "SIT 2x" = "Release 2×")) +

theme_classic() + theme(legend.position = c(0.2,0.1))

# Hatch cumulative effect

effect_plot_hatch_phase <- bind_rows(sit1x_hatch, sit2x_hatch) %>%

drop_na(cum.effect) %>% mutate(release_phase = factor(release_phase, levels = c("SIT 1x","SIT 2x"))) %>%

ggplot(aes(x = date, fill = release_phase, color = release_phase)) +

geom_ribbon(aes(ymin = cum.effect.lower, ymax = cum.effect.upper), alpha = 0.2) +

geom_vline(xintercept = ymd(c("2020-10-27","2021-07-12")), color = "darkred", linetype = INT_LTY, linewidth = 0.8) +

geom_line(aes(y = cum.effect, group = 1)) + geom_hline(yintercept = 0) +

labs(x = "Month/Year", y = "Cumulative Causal Effect") +

scale_y_continuous(expand = c(0,0)) + scale_x_date(date_breaks = "10 week", date_labels = "%m/%y") +

scale_color_manual(name = "", values = c("SIT 1x" = MAIN_1, "SIT 2x" = MAIN_2), labels = c("SIT 1x" = "Release 1×", "SIT 2x" = "Release 2×")) +

scale_fill_manual(name = "", values = c("SIT 1x" = MAIN_1, "SIT 2x" = MAIN_2), labels = c("SIT 1x" = "Release 1×", "SIT 2x" = "Release 2×")) +

theme_classic() + theme(legend.position = c(0.2,0.1))

causal_impact_plot_sit1 <- ggarrange(

gg_original_eggs_1, gg_original_eggs_2, effect_plot_eggs_phase,

gg_original_hatch_1, gg_original_hatch_2, effect_plot_hatch_phase,

gg_original_adult_1, gg_original_adult_2, effect_plot_adult_phase,

ncol = 3, nrow = 3, common.legend = TRUE, legend = "bottom", labels = "AUTO", align = "hv"

)

# Save causal figures

causal_plot_1x <- ggarrange(impact_1_plot, egg_impact_1_plot, hatch_impact_1_plot, labels = "AUTO", ncol = 3, nrow = 1)

causal_plot_2x <- ggarrange(impact_2_plot, egg_impact_2_plot, hatch_impact_2_plot, labels = "AUTO", ncol = 3, nrow = 1)

# =========================================================

# 9) GLMMs for ETD, hatch, induced sterility (EMMeans)

# =========================================================

phases_primary <- c("baseline","PPF","BTI","SIT 1x","SIT 2x")

etd_dat <- ovitrap %>% filter(phase %in% phases_primary, !is.na(ETD), ETD > 0)

mod_etd <- glmmTMB(ETD ~ location * phase + (1|year), data = etd_dat, family = Gamma(link = "log"), weights = w)

hatch_dat2 <- ovitrap %>% filter(phase %in% phases_primary, !is.na(hatch_rate)) %>% mutate(hatch_rate = pmin(pmax(hatch_rate, 1e-4), 1-1e-4))

mod_hatch2 <- glmmTMB(hatch_rate ~ location * phase + (1|year), data = hatch_dat2, family = beta_family(link = "logit"), weights = w)

ster_dat2 <- ovitrap %>% filter(phase %in% c("SIT 1x","SIT 2x"), !is.na(induced_sterility)) %>%

mutate(ind_st = induced_sterility, ind_st = ifelse(ind_st > 1 & ind_st <= 100, ind_st/100, ind_st), ind_st = ifelse(ind_st < 0 | ind_st > 1, NA_real_, ind_st), ind_st = pmin(pmax(ind_st, 1e-4), 1-1e-4)) %>%

drop_na(ind_st)

mod_ster2 <- glmmTMB(ind_st ~ location * phase + (1|year), data = ster_dat2, family = beta_family(link = "logit"), weights = w)

# ETD — EMMs per location

e_etd <- emmeans(mod_etd, ~ phase | location) %>% regrid() %>% as.data.frame() %>%

transmute(location, phase, value = fmt_ci(response, asymp.LCL, asymp.UCL, digits = 2))

# Hatch — EMMs per location

e_hr <- emmeans(mod_hatch2, ~ phase | location) %>% regrid() %>% as.data.frame() %>%

transmute(location, phase, value = fmt_ci(response, asymp.LCL, asymp.UCL, digits = 1, percent = TRUE))

# Induced sterility — EMMs per location (SIT phases only)

e_is <- emmeans(mod_ster2, ~ phase | location) %>% regrid() %>% as.data.frame() %>%

transmute(location, phase, value = fmt_ci(response, asymp.LCL, asymp.UCL, digits = 1, percent = TRUE))

# Sterile:Wild ratio summary (geometric mean ± 1 SD on log scale)

ratio_summary <- ratio_week %>%

filter(release_phase %in% c("SIT 1x","SIT 2x")) %>%

group_by(location, release_phase) %>%

summarise(

n_pos = sum(sterile_wild > 0),

mean_log = mean(log1p(sterile_wild[sterile_wild > 0]), na.rm = TRUE),

sd_log = sd(log1p(sterile_wild[sterile_wild > 0]), na.rm = TRUE),

.groups = "drop"

) %>%

mutate(

geom_mean = exp(mean_log) - 1,

geom_low = exp(mean_log - sd_log) - 1,

geom_high = exp(mean_log + sd_log) - 1,

value = sprintf("%.2f ± %.2f", geom_mean, pmax(geom_high - geom_mean, 0))

)

# =========================================================

# 10) Final Table (Table1_BT) — by area and parameter

# =========================================================

ensure_cols <- function(df, cols) {

for (cc in cols) if (!cc %in% names(df)) df[[cc]] <- NA_character_

df

}

make_tbl <- function(df, area_label, param_label) {

df %>%

dplyr::filter(location == tolower(area_label)) %>%

dplyr::transmute(Parameter = param_label, area = area_label, phase, value) %>%

tidyr::pivot_wider(names_from = phase, values_from = value)

}

PHASE_COLS <- c("baseline","PPF","BTI","SIT 1x","SIT 2x")

# ETD

tbl_etd <- dplyr::bind_rows(

make_tbl(e_etd, "Brasilia", "ETD"),

make_tbl(e_etd, "Pina", "ETD")

) %>%

ensure_cols(PHASE_COLS) %>%

dplyr::select(Parameter, area, dplyr::all_of(PHASE_COLS))

# Hatch rate

tbl_hatch <- dplyr::bind_rows(

make_tbl(e_hr, "Brasilia", "HR(%)"),

make_tbl(e_hr, "Pina", "HR(%)")

) %>%

ensure_cols(PHASE_COLS) %>%

dplyr::select(Parameter, area, dplyr::all_of(PHASE_COLS))

# Induced sterility

tbl_is <- dplyr::bind_rows(

make_tbl(e_is, "Brasilia", "IS(%)"),

make_tbl(e_is, "Pina", "IS(%)")

) %>%

ensure_cols(PHASE_COLS) %>%

dplyr::select(Parameter, area, dplyr::all_of(PHASE_COLS))

# ST:WT ratio

ratio_fmt <- ratio_summary %>%

dplyr::select(location, release_phase, value) %>%

dplyr::mutate(Parameter = "ST:WT",

area = dplyr::if_else(location == "brasilia","Brasilia","Pina")) %>%

dplyr::select(Parameter, area, release_phase, value) %>%

tidyr::pivot_wider(names_from = release_phase, values_from = value) %>%

ensure_cols(PHASE_COLS) %>%

dplyr::select(Parameter, area, dplyr::all_of(PHASE_COLS))

# Final Table

Table1_BT <- dplyr::bind_rows(tbl_etd, tbl_hatch, tbl_is, ratio_fmt) %>%

dplyr::arrange(match(Parameter, c("ETD","HR(%)","IS(%)","ST:WT")), dplyr::desc(area))

readr::write_csv(Table1_BT, "table1_BT.csv")

print(Table1_BT)
